# Wetter farming: raising water table and biochar for reduced GHG emissions while maintaining crop productivity in agricultural peatlands

**DOI:** 10.1007/s42773-025-00487-7

**Published:** 2025-09-15

**Authors:** Peduruhewa H. Jeewani, Emmanuella Oghenefejiro Agbomedarho, Chris D. Evans, David R. Chadwick, Davey L. Jones

**Affiliations:** 1 School of Environmental and Natural Sciences, Bangor University, Bangor, Gwynedd LL57 2UW UK; 2 UK Centre for Ecology & Hydrology, Bangor, Gwynedd LL57 2UW UK

**Keywords:** Peat, Biochar, Water table management, Nutrient cycling, Greenhouse gas emission, Lettuce biomass

## Abstract

Despite their high agricultural productivity, drained and cultivated peats are highly susceptible to degradation and significant sources of greenhouse gas (GHG) emissions. This study investigates the potential of water table manipulation and biochar application to mitigate GHG losses from agricultural peats. However, balancing the need for agricultural production with securing the ecosystem function of the peat under high water table (WT) conditions poses a significant challenge. Therefore, we grew lettuce in a controlled mesocosm experiment with either a high (HW) or low (LW) water table and monitored emissions of CO_2_, CH_4_ and N_2_O over 4 months using a mesocosm method. Concurrent measurements of soil solution, plant measurements and microbial sequencing allowed identification of the key controls on GHG emissions. Raising the WT significantly reduced CO_2_ emissions (18%), and N_2_O emission (40%), but eventually increased CH_4_ emission (2.5-fold) compared to the Control + LW. Biochar amendment with raised WT provided the strongest reduction in CO_2_ equivalent GHG emission (4.64 t CO_2_eq ha^−1^ yr^−1^), compared to Control + LW. We found that biochar amendment modified the microbial community composition and diversity (Shannon index 8.9–9.3), lowering the relative abundance of peat decomposers (such as Ascomycota). Moreover, biochar amendments produced 38–56% greater lettuce biomass compared to the unamended controls, irrespective of water table level, suggesting that biochar application could generate economic benefits in addition to reduced GHG emissions. Mechanisms responsible for these effects appeared to be both abiotic (e.g. via effects of the biochar physicochemical composition) and biotic via changing the soil microbiome. Overall, the combination of high-water table and biochar amendment enhanced total soil C, reduced peat decomposition, suppressed CH_4_ and N_2_O emissions, and enhanced crop yields.

**Supplementary Information:**

The online version contains supplementary material available at 10.1007/s42773-025-00487-7.

## Highlights

- Raising groundwater levels of an agricultural peat reduced CO_2_ emissions by 18%.
- Raising water levels with biochar reduced GHG emissions by 4.64 t CO_2_eq ha^−1^ yr^−1^.
- Biochar addition increased lettuce yield (38−56%) irrespective of groundwater level.
- Biochar addition reduced the relative abundance of peat decomposers (Actinobacteria and Ascomycota).

**Supplementary Information:**

The online version contains supplementary material available at 10.1007/s42773-025-00487-7.

## Introduction

Draining peats for agriculture causes the carbon (C) that was locked up in the peat to oxidise and be released into the atmosphere as CO_2_. Approximately 10% of global peats are used for agriculture, yielding some of the world's most productive soils when drained and cultivated (Leifeld and Menichetti 2018). These drained peats contribute significantly to anthropogenic greenhouse gas (GHG) emissions, accounting for approximately 4% (2 Gt CO_2_ equivalent CO_2_eq yr^−1^) (Joosten et al. 2016). A recent study highlights the significant contribution of drained peats to global warming, suggesting that their emissions between 2020 and 2100 could consume 12–41% of the remaining C budget needed to limit global warming to 1.5–2 °C (Leifeld et al. 2019). By restoring the water table and re-wetting the peat, the remaining C in the peat can be protected and GHG emissions significantly reduced. However, at the present time very few food crops can be produced on peat with a high-water table. Reconciling the need to preserve agricultural peat for climate change mitigation with the demand for food production presents a significant challenge.

Agricultural peats are the dominant source of national GHG emissions from peat (Tiemeyer et al. 2016; Evans et al. 2017; Leifeld and Menichetti 2018). Within these areas, the depth of peat has been declining by between 0.3 and 3 cm yr^−1^ (Dawson et al. 2010), and it has been reported that 35–100% of peat subsidence is attributable to microbial mineralization processes (Leifeld et al. 2011). While methane (CH_4_) consumption in these soils is limited, N_2_O emissions are substantial, accounting for approximately one-third to one-half of the total GHG emission, significantly impacting their overall climate impact (Taft et al. 2018). Peatland rewetting reduces oxidative C loss in arable areas and is considered a cost-effective method to curb GHG emissions (Leifeld et al. 2019; Evans et al. 2021). However, raising water levels near the surface may increase CH₄ emissions (Evans et al. 2021; McNicol et al. 2023). In general, it appears that raising water levels reduces N_2_O emissions (Prananto et al. 2020), although there remains a risk that intermediate or fluctuating water levels, combined with ongoing fertilisation for crop production, could lead to elevated N_2_O emissions (Wang et al. 2024). Given that CH_4_ and N_2_O have a greater radiative forcing impact than CO_2_, there is a risk that measures to reduce CO_2_ emission from peat could generate a short-term warming impact, although given the longer atmospheric lifetime of CO_2_ it is generally accepted that conservation of peat C stocks through re-wetting will generate climate benefits over longer time horizons (Gunther et al. 2020).

Whilst raising water levels is necessary to preserve peat C, it can create anoxic conditions near the root zone, hindering root growth and depth, and consequently reducing crop yields (Wen et al. 2019). For example, when the groundwater table was raised, wheat, rye, and vetch cover crops experienced yield losses of 22%, 29%, and 25%, respectively. (Wen et al. 2019; Evans et al. 2023). At the core of the challenge for sustainable management of agricultural peat is that the vast majority of crops grown were developed for dryland conditions and are therefore unsuitable for cultivation under wetland conditions (Freeman et al. 2022). Crucially, very few food crops can be grown under ‘paludiculture’ conditions in high-latitude peat (Page et al. 2020), and converting land currently used for food production to other uses such as biomass crops or energy production therefore risks simply displacing the environmental consequences of food production, including GHG emissions and habitat loss, to other areas.

Solving this seemingly intractable ‘food versus carbon’ problem in agricultural peat requires novel solutions. Biochar emerged as a promising "carbon-negative" solution, initially proposed as a soil amendment to promote soil carbon sequestration (Zhang et al. 2010), potentially at millennial timescales. Its C stabilization is due to high biochemical stability against microbial decomposition (Yin et al. 2022), which may be further enhanced under waterlogged conditions. Biochar may also exert a suppressive effect on the decomposition of soil organic carbon (SOC), as well as the production of non-CO_2_ GHGs (Jeewani et al. 2025). While numerous studies have reported reductions in GHG emissions following biochar application, others have found neutral effects (Lyu et al. 2022) or even increases under specific conditions, such as anaerobic soils or biochars with high volatile matter content (Yin et al. 2022; Ma et al. 2023). The potential of biochar to improve crop growth has been widely explored in dryland production systems, e.g. for maize and soybean (Palansooriya et al. 2019; Hou et al. 2022), but has yet to be trialled in agricultural peat. Several studies have reported no significant yield increase or even yield reductions, particularly at high application rates or in already fertile soils (Biederman and Harpole 2013). In this study we therefore investigated the possibility of biochar application, with and without water table manipulation, to mitigate GHG emissions, modify the soil biogeochemical and microbial environment, and maintain the yields of a high-value food crop (lettuce, *Lactuca sativa* L.) in an agricultural peat. We hypothesised that: (1) High water tables would suppress CO₂ emissions but increase CH₄ emissions due to enhanced methanogenesis; (2) High water tables would reduce lettuce biomass and root biomass due to anaerobic root zone conditions; and (3) Biochar application combined with high water tables would suppress GHG emissions but lower lettuce biomass, while biochar with low water tables would maintain higher biomass through increased root biomass, though with less GHG mitigation.

## Materials and methods

### Study site and experimental design

The soil was sampled from a site in East Anglia, UK (52^o^31’N,0^o^23’E). It has a mean annual temperature of 13 °C and mean annual rainfall of around 600 mm (Taft et al. 2018). The site is a flat, drained lowland peat with an organic layer approximately 1.5 m deep, which was drained in 1940 (Musarika et al. 2017). It is managed as a high-value horticultural rotation (e.g. lettuce, celery, radish) with cereal break crops such as wheat and maize. The soil is classified as an Earthy Sapric Fen Soil (USDA Soil Taxonomy system). Soil properties are detailed in Table 1.

**Table 1:** Soil and biochar characteristics

Soil characteristic	Lowland peat	Biochar	
Total carbon (C) (%)	27.6 ± 2.6	73.76 ± 2.6	
Total nitrogen (N) (%)	1.81 ± 0.45	0.4 ± 0.06	
Hydrogen (%)	–	4.1 ± 0.2	
Atomic H/C	–	0.66 ± 0.12	
SPAC (%)	–	25	
C:N ratio	16.0 ± 4.8	184	
Organic matter content (%)	50.3 ± 1.8	–	
pH (H_2_O)	6.54 ± 0.05	5.65 ± 0.07	
Electrical conductivity (EC) (μS cm^−1^)	200 ± 4	51.53 ± 3.8	
Bulk density (BD) (g cm^−^^3^)	0.52 ± 0.05	–	
NO_3_^−^ (mg L^−1^)	4.05 ± 0.29	–	
NH^+^_4_ (mg L^−1^)	4.48 ± 0.22	–	
SO^2−^_4_ (mg L^−1^)	1.35 ± 0.42		–
PO^3−^_4_ (mg L^−1^)	1.05 ± 0.11		–

All values mean ± standard errors (n = 4). Where applicable, the data is expressed on a dry weight basis

To determine the potential synergistic effects of water table manipulation and biochar amendments, we designed a mesocosm experiment on the outdoor open space at School of Natural Sciences, Bangor University. Soil mesocosm preparation was performed in May 2023. Approximately ten soil samples representative of marginally degraded agricultural site were taken from the upper 0.2 m of the profile and passed through a 5 mm aperture sieve, to remove larger stones, debris and vegetation. Samples were bulked together to make a single sample to minimise within-soil variation and repacked according to its field bulk density.

Sixteen mesocosms were arranged using PVC pipes with a height of 30 cm and an inner diameter of 20 cm. To maintain the target water table level within the mesocosms throughout the experiment, they were placed in modified outer containers and topped naturally with rainwater. After a week of acclimation, the water table in the mesocosms was raised to − 10 cm (noted as HW) and in other mesocosms the level was kept at − 15 cm from the soil surface (noted as LW). Evans et al. (2021) found that shallow water tables (around − 10 cm to − 15 cm) are critical for minimizing CO₂ emissions, as deeper drainage increases aerobic decomposition of peat. Meanwhile, these depths also help limit CH₄ emissions, which rise sharply at shallower (near-surface or above-surface) water levels. To prevent deviations caused by rainfall, the outer containers were punctured at precise heights, allowing for controlled drainage without compromising the desired water table levels. For each water table level, four mesocosms were amended with biochar in an equivalent total C loading rates of 10 t of C ha^−1^, while the other four mesocosms were left unamended. All the mesocosms were planted with lettuce (*Lactuca sativa* L.) seedlings (6 cm tall uniform seedlings). The experimental design consisted of four distinct treatments, each replicated four times, thus resulting in a total of 16 experimental units. The treatments were: (1) biochar incorporated (15.8 g of biochar) into 0–10 cm soil layer at low water table depth (Biochar + LW), (2) biochar incorporated and maintained at high water table depth (Biochar + HW), (3) no biochar with low water table depth (Control + LW), and (4) no biochar with highwater table depth (Control + HW). The experiment was conducted for 110 days with two successive lettuce crops, in line with the typical management of lettuce crops at the farm from which soil was collected (Fig. S3). The biochar utilized for the experiment was derived from chippings of the stems from the bioenergy crop *Miscanthus* (pyrolyzed at 450 °C) and consisted of particles with a size of < 10 mm (Table 1). Miscanthus biochar was chosen due to its high lignin content and uniform woody biomass which creates a structurally stable biochar, while the crop itself offers additional environmental benefits through its low-input perennial growth habit and high C sequestration potential (Zub and Brancourt–Hulmel 2010). Pyrolysis at ∼450 °C intermediate temperature produces a biochar with a balanced combination of surface area, porosity, and functional groups that enhance its stability and interaction with soil nutrients and contaminants (Keiluweit et al. 2010). The selected particle size (< 10 mm) ensures better mixing and distribution in soil matrices, promoting improved biochar–soil interactions and more uniform effects on soil properties with a larger surface area- to -volume ratio (Downie et al. 2009).

### Greenhouse gas emission measurements

Fluxes of CO₂, CH₄, and N₂O were measured using mesocosms equipped with cylindrical, opaque PVC chambers that were securely sealed during each measurement event. The headspace was 10 cm high and 20 cm in diameter, and the top was fitted with a Suba–Seal^®^ (Sigma–Aldrich Poole, UK) for gas collection via a syringe and needle. When sampling, the cap was fitted tightly over the top of the mesocosm, and wax was used to seal the connection between the cap and the mesocosm to ensure airtightness and prevent gas leakage. We measured GHG (CO_2_, N_2_O, CH_4_) fluxes for 110 days with intensive sampling (once every 3 days for the first 60 days) followed by biweekly sampling. At each sampling event, three gas samples were taken from the 1.57 − l headspace of each chamber at 0, 30, and 60 min. These samples were then stored in pre-evacuated 20 ml glass vials. Gas analysis was performed using a gas chromatograph equipped with an electron capture detector (ECD) for N_2_O and a flame ionization detector (FID) with a methanizer for CH_4_ and CO_2_. A TurboMatrix 110 autosampler (PerkinElmer Inc., Shelton, CT, USA) was used to automate the sample handling process. Gaseous fluxes were calculated based on the changes in headspace gas concentrations, taking into account the air temperature, headspace volume, and soil area (Wen et al. 2019). The headspace GHG concentrations were checked for the linearity (R^2^ ≥ 0.90, *n* = 4) by taking additional headspace measurements (T0, T30 and T60) from mesocosms on each sampling occasion.

1 $$F = \frac{{{\Delta }c}}{{{\Delta }t}} \times AV \times R \times TP$$

where:

***F*** = Gas flux (e.g., µmol m^−2^ s^−1^ or mg m^−2^ h^−1^).

***Δc/Δt*** = Rate of change in gas concentration over time (slope of concentration vs. time).

***V*** = Volume of the chamber (m^3^).

***A*** = Surface area covered by the chamber (m^2^).

***P*** = Atmospheric pressure (Pa).

***R*** = Universal gas constant (8.314 J mol^−1^ K^−1^).

***T*** = Temperature in Kelvin (K).

GHG emissions were calculated by subtracting the gas concentrations at time 0 from those measured 60 min later, with adjustments made for temperature and the ratio of chamber volume to soil surface area. Cumulative emissions of CO_2_, N_2_O and CH_4_ were calculated by linear interpolation of measured flux rates (Wen et al. 2019).

2 $${\text{Cumulative emissions }} = \,\sum {\_i^{ \wedge } n} \,\left[\kern-0.15em\left[ {\left( {\left( {R\_\left( {i - 1} \right) + R\_i} \right)/2 \times Di} \right)} \right]\kern-0.15em\right]$$

where *R*_*i−*1_ and *R*_*i*_ are the rate of GHG flux in the *i − *1 and *i*th sampling, *D*_i_ is the number of days between *i − *1 and *i*th sampling and *n* is the number of sampling times. To allow comparison among treatments, GHG emissions were converted to CO_2_ equivalents (CO_2_eq) based on 100‐yr global warming potential conversion factors of 273 for N_2_O and 27 for CH_4_ (IPCC AR6, 2024).

3 $${\text{Total}}\,{\text{GHG}}\,{\text{emissions}}\,\left( {{\text{CO}}_{{2 }} {\text{equivalent}}} \right)\,{ = }\,{\text{CO}}_{{2}} \,{ + }\,\left( {{273}\, \times \,{\text{N}}_{{2}} {\text{O}}} \right)\,{ + }\,\left( {{27 }\, \times \,{\text{CH}}_{{4}} } \right)$$

### Soil solution parameters

On each sampling occasion, soil solutions were collected non-destructively using Rhizon-MOM^®^ samplers (Rhizosphere Research Products, Netherlands). Rhizon-samplers were inserted at 10 cm depth horizontally into the repacked soil through the side of the columns and remained in-situ throughout. Soil solution was collected by connecting the Rhizon sampler to pre evacuated 9 ml tubes (Vacu test, Italy), on dates as close as possible to those when GHG sampling took place (once every 3 days for the first 60 days, followed by biweekly sampling). Soil solution samples were stored at − 20 °C in sterile vacutainers^®^ (Elkay Laboratory Products, UK) and later analysed for dissolved organic carbon (DOC), dissolved organic nitrogen (DON), NO_3_^−^ and NH_4_^+^ concentration. DOC and DON were measured using a Multi N/C 2100/2100 analyser (AnalytikJena AG, Jena, Germany). NH_4_^+^ and NO_3_^−^ were measured by spectrophotometry on a PowerWave-XS microplate reader using the colorimetric methods described in Mulvaney (1996) and Miranda et al. (2001), respectively. NH_4_^+^ and NO_3_^−^ concentrations were determined spectrophotometrically using a PowerWave-XS microplate, colorimetric methods adapted from Mulvaney (1996) and Miranda et al. (2001) respectively.

### Lettuce growth and biomass measurements

The lettuce plants were harvested twice; the first harvest was 60 days after seedling planting, after which a second seedling was planted and harvested 50 days later (110 day experimental duration in total). We destructively sampled the plants at harvest and carefully collected the roots. Fresh biomass for roots and shoots was measured immediately. Dry weights of roots and shoots were measured by oven drying (48 h, 80 °C).

### DNA extraction and 16S rRNA gene metabarcoding

A Zymo research soil DNA isolation kit (Zymo Research, USA) was used to extract DNA from soil (0–10 cm). DNA concentration and purity were determined using a NanoDrop spectrophotometer (Thermo Scientific). Subsequent PCR amplifications were conducted on an ABI 9700 PCR instrument (Thermo Fisher Scientific, Waltham, MA, USA). The fragment length of the bacterial 16S V3–V4 amplification region was 450–550 bp and the primers were 341F and 806R. Primer sequences were CCTAYGGGRBGCASCAG and GGACTACNNGGGTATCTAAT. For the fungal community measurements, the amplification region was ITS2 (380 bp) with the primers of ITS3–ITS4 including primer sequences of GCATCGATGAAGAACGCAGC and TCCTCCGCTTATTGATATGC. The PCR reaction was performed in a 25 μl mixture containing 12.5 μl of 2xTaq Plus Master Mix, 3 μl of 2 ng μl^ − 1^ BSA, 1 μl of 5 μM Forward Primer, 1 μl Reverse Primer (5 μM), 2 μl template DNA, and 5.5 μl ddH_2_O. The PCR amplification was performed using the following cycling conditions: an initial denaturation step at 95 °C for 5 min, followed by 28 cycles of denaturation at 95 °C for 45 s, annealing at 55 °C for 50 s, and extension at 72 °C for 45 s. A final extension step was conducted at 72 °C for 10 min, followed by cooling and storage at 4 °C. PCR products were visualized on a 1% agarose gel and subsequently purified using the Agencourt AMPure XP kit. Paired-end sequencing of the purified products was conducted on an Illumina MiSeq PE300 platform. Bacterial and fungal DNA amplification and sequencing were performed by Novogene Co. Ltd., Beijing, China.

### Statistical analysis

Data normality was assessed using the Shapiro–Wilk test, and homogeneity of variances was evaluated with Levene's test. To meet the assumptions of normality and homoscedasticity, data were transformed using square root or logarithmic transformations as needed. Treatment effects were analyzed using one-way analysis of variance (ANOVA). Pairwise comparisons between treatment means were conducted using Tukey's post hoc tests with appropriate adjustments for multiple comparisons. All analyses were conducted using a significance level of *p* < 0.05. Only statistically significant results are discussed. SPSS Statistics 24 (IBM Corp., NY, USA) was the primary software used for statistical analyses. Data are presented as mean ± SE (standard error). From the 16S and ITS data, alpha diversity was calculated using the Shannon index on raw OTU abundance tables after filtering out contaminants. The significance of diversity differences between location treatments was tested using an ANOVA model followed by a post-hoc Tukey HSD test. Distance-based linear model multivariate analysis (distLM) was conducted in a distLM_forward3 software (Anderson 2003) to determine the relative treatment effects on physiochemical variables such as pH, NH_4_^+^ concentration, moisture content, DON, DOC, redox potential and root and shoot biomass. Origin 2022 (Origin Lab, Northampton, MA, USA) and RStudio version 1.0.143 (http://www.rstudio.com/) were used for data visualization.

## Results

### Effect of water table depth and biochar amendment on greenhouse gas emission

**Fig. 1:**
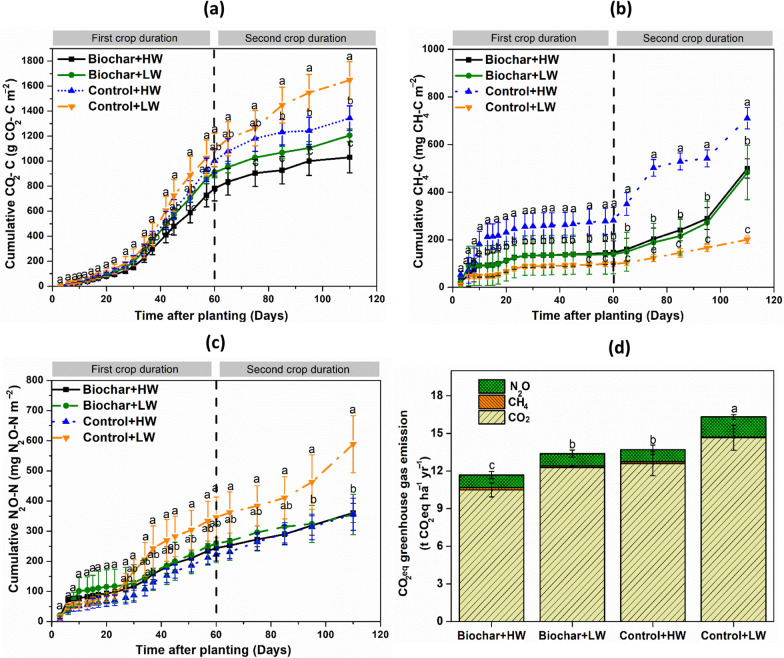
Effect of biochar amendments and water table management (HW −10 cm, and LW −15 cm from the soil surface) on cumulative soil CO_2_ (**a**), CH_4_ (**b**), N_2_O (**c**) and CO_2_ equivelent greenhouse gas emission (**d**) from an agricultural peat soil under lettuce production. The biochar amendments included *Miscanthus* biochar. Values represent means ± standard errors (*n* = 4). LW indicates water table level at −15 cm depth; HW indicates water table level at −15 cm depth. Treatments with different letters are significantly different (Tukey, *p* < 0.05)

During the 110-day experimental period, the highest cumulative CO_2_ emission was reported in the Control + LW treatment (Fig. 1a and Table S1). Cumulative CO_2_ emissions in the high-water table treatment (Control + HW) were 303.75 g CO_2_ − C m^−2^ (*p* < 0.01) lower than in the Control + LW (Fig. 1a). Biochar addition had a strong negative effect on cumulative CO_2_ emissions, to the extent that emissions from the Biochar + HW treatment were 24% lower than those from the Control + HW treatment (i.e. the suppressive effect of biochar addition exceeded that of raised water levels). The lowest measured CO_2_ emissions were from the Biochar + HW treatment, with cumulative emissions 615.84 g CO_2_ − C m^−2^ (*p* < 0.01) lower than those from the Control + LW treatment.

Cumulative CH_4_ emissions were lower in the Control + LW treatment, around 70% lower than in the Control + HW treatment (Fig. 1b). Biochar addition to both water level treatments suppressed cumulative CH_4_ emission by around 25% compared to the Control + HW treatment, but emissions remained higher than those from the Control + LW treatment. Cumulative N_2_O emissions were highest from the Control + LW treatment, but similar (around 35% lower than Control + LW) for all other treatments (Fig. 1c). No significant differences were found between the two biochar addition treatments.

Based on 100-year global warming potentials for CH_4_ and N_2_O, overall GHG emissions were highest for the Control + LW treatment (16.6 ± 2.0 t CO_2_eq ha^−1^ over the 110-day experimental period), followed by the Control + HW treatment (13.7 ± 1.3) (Fig. 1d). Cumulative emissions were lower for both biochar added treatments, and lowest from Biochar + HW treatment (11.7 ± 1.6 t CO_2_eq ha^−1^, versus 13.4 ± 1.0 t CO_2_eq ha^−1^ for Biochar + LW). Under high water table conditions, biochar treatments significantly decreased net GHG emissions relative to the controls. Across all treatments, CO_2_ comprised > 90% of total net GHG emissions, followed by N_2_O, while CH_4_ made a negligible contribution (< 0.5%). Note that our calculations do not incorporate the input of C to the peat in biochar, which could be considered a ‘negative emission’, but that any CO_2_ released via oxidation of this biochar would have been captured as part of the total measured CO_2_ emission.

### Soil solution dynamics

**Fig. 2:**
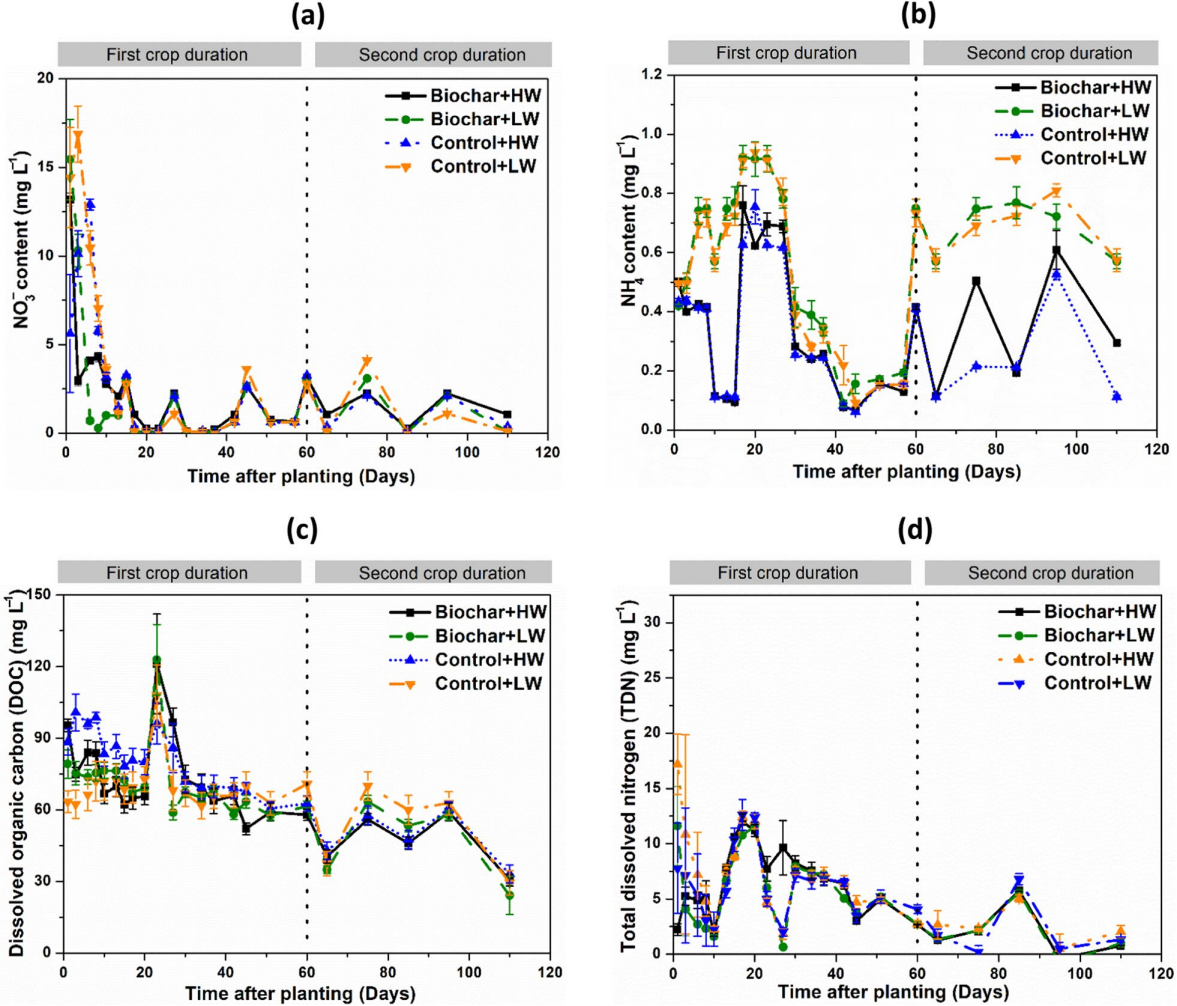
Effect of biochar amendments and water table management (HW − 10 cm, and LW − 15 cm from the soil surface) on the temporal variation of NO_3_^−^ − N (**a**), NH_4_^+^ − N (**b**), dissolved organic C (**c**), and total dissolved nitrogen (**d**), in soil solution of an agricultural peat soil under lettuce production. The biochar amendments included *Miscanthus* biochar. Values represent means ± standard errors (*n* = 4). LW indicates water table level at − 15 cm depth; HW indicates water table level at 15 cm depth

Soil solution NO_3_^−^ concentrations decreased substantially until day 20, after which concentrations remained low (Fig. 2a). Soil solution NH_4_^+^ content in all treatments ranged from 0.2 to 1 mg N L^−1^, and there were no significant differences during the first 60 days (Fig. 2b). However, between days 60 and 110 (i.e. the second lettuce cropping period) soil solution NH_4_^+^ content was higher in the low water table treatments compared to high water table treatments (*P* < 0.05; Fig. 2b). DOC content slightly decreased in all treatments throughout the experiment (Fig. 2c). DON concentrations of all treatments decreased substantially until day 20, and then remained low (Fig. 2d). No significant differences in DOC and DON concentrations were observed between treatments during the lettuce growth period.

### Lettuce shoot and root biomass

**Fig. 3:**
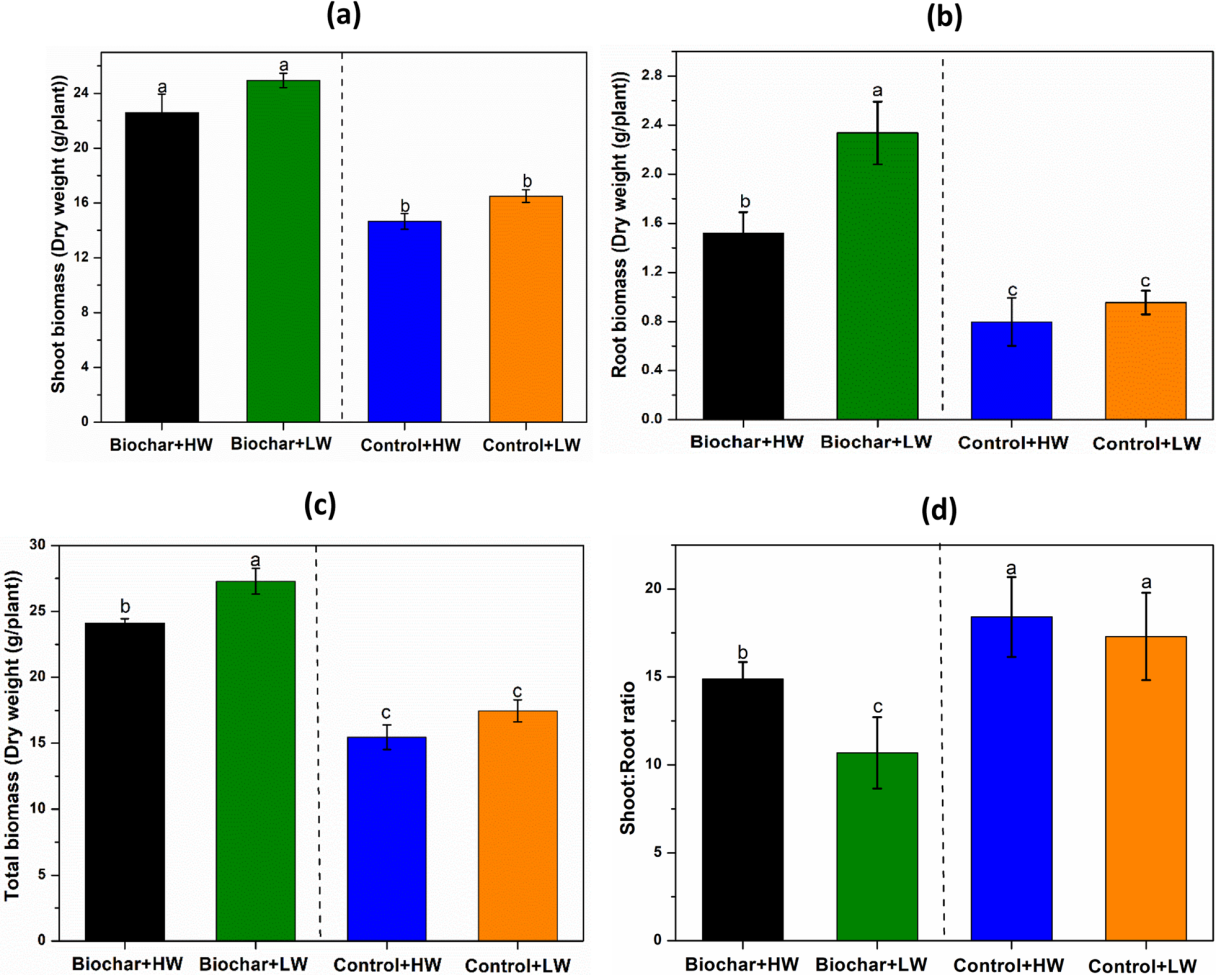
Effect of biochar amendments and water table management (HW − 10 cm, and LW − 15 cm from the soil surface) on the growth parameters of lettuce plants growing in an agricultural peat soil: **a** Shoot Biomass; **b** Root Biomass; **c** Total Biomass; and **d** Root: shoot ratio. Values represent means ± standard error (*n* = 4). Treatments with different letters are significantly different (Tukey, *p* < 0.05)

Lettuce shoot and root biomass was significantly influenced by biochar addition compared to controls (Fig. 3a, b respectively). Dry shoot biomass increased by 22% and 21% for Biochar + HW and Biochar + LW, respectively, compared to the respective controls without biochar (Fig. 3b). The dry root biomass in the biochar treatments increased by 8.44 g (Biochar + HW) and 8.08 g (Biochar + HW) compared to the respective controls (Fig. 3b). At the same time, the root to shoot ratio (based on dry biomass) varied between 14.8 and 18.4 (Fig. 3d), with significantly lower values in both biochar-amended treatments compared to the no-biochar controls, and lower values in the Biochar + LW treatment compared to the Biochar + HW treatment.

### Microbial community response

**Fig. 4:**
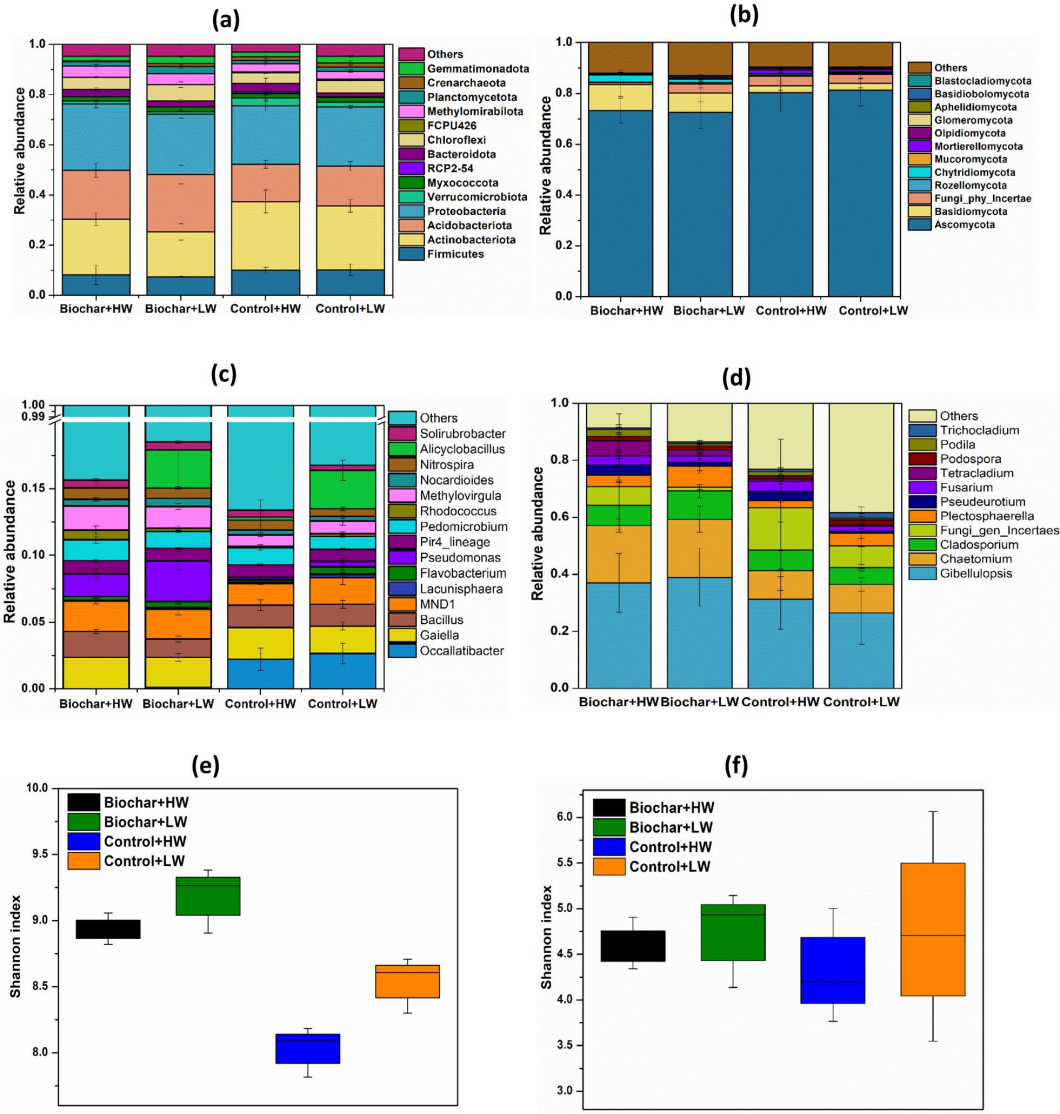
Effect of biochar amendments and water table management (HW − 10 cm, and LW − 15 cm from the soil surface) on bacterial and fungal community dynamics in an agricultural peat soil. **a** and **b** illustrate the relative abundance of bacterial and fungal at phylum level, **c** and **d** illustrate the relative abundance of bacterial and fungal at genus level, **e** and **f** represent alpha diversity of soil bacterial and fungal communities, respectively. The Shannon index was calculated with all OTUs. The horizontal bars within boxes represent the median. The tops and bottoms of boxes represent 75th and 25th quartiles, respectively (*n* = 4). Treatments with different letters are significantly different (Tukey, *p* < 0.05)

The changes observed in relative abundances of bacterial phyla and genera in four treatments were associated with water table level as well as biochar amendments (Fig. 4). The predominant bacterial phyla in soils were Firmicutes, Actinobacteria, Acidobacteria, Proteobacteria, Gemmatimonadetes, Chloroflexi and Bacteroidetes. These taxa accounted for 89% of the bacterial sequences in all treatments (Fig. 4a). As shown in Fig. 4a, the relative abundance of Acidobacteria increased in biochar added treatments, while Proteobacteria and Actinobacteria were higher in controls. Ascomycota were the most abundant fungal phylum across all the treatments, with relative abundances from 72% to 80%. The abundance of Basidiomycota was significantly increased by biochar amendment, by 10.3% in Biochar + HW and 7.3% in the Biochar + LW treatment.

At genus level, the relative abundance of several genera differed significantly between treatments. The controls had significantly higher relative abundance of *Occallatibacter* than the biochar treatments (Fig. 4c). Similarly, the relative abundance of genera *Pseudomonas* was 10 times higher in biochar added treatment compared to controls. Moreover, LW treatments increased the abundance of the genus *Alicyclobacillus* by (2.8 ± 0.02)% compared to HW treatments, regardless of biochar amendments. Fungal genera of *Gibellulopsis* and *Chaetomium* showed significantly higher abundances in the biochar-amended soils in comparison to the unamended controls.

Shannon’s diversity revealed depletion of bacterial and fungal similarity in Control + LW and Control + HW compared to Biochar + LW and Biochar + HW (Fig. 4e, f). The Shannon index of bacteria was highest in biochar added treatments and lowest in Control + HW (Fig. 4e). The fungal Shannon index ranged from 3.5 to 6.0 across all treatments (Fig. 4f).

Best multivariate distance-based linear modeling (distLM) analysis (Anderson and Legendre 1999) was used to analyze the effects of biogeochemical factors including pH, DOC, EC, NO_3_^−^, NH_4_^+^, root biomass, C/N ratio, TC and redox potential on the microbial community (Table 2). The soil bacterial community was related to pH (8.2%), DOC (7.9%), soil moisture (7.1%), NO_3_^−^ (6.4%), root biomass (5.3%) and C/N ratio (4.2%). The soil fungal community was related to NH_4_^+^(10.1%), soil moisture content (8.5%) and TC (8.9%). In mesocosms with FeSO_4_ added the strongest observed relationship was with SO_4_^2−^ concentration (13.5%).

**Table 2:** Contributions of soil and plant variables to shaping the bacterial and fungal community based on Bray–Curtis dissimilarities analyzed by distance-based linear modeling (distLM) analysis

Soil and plant variable	Contribution to Bacteria %	Soil and plant edaphic variable	Contribution to Fungi %
pH	8.2*	NH_4_^+^	10.1**
DOC	7.9*	TC	8.9*
Soil moisture content	7.1*	Soil moisture content	8.5**
NO_3_^−^	6.4*	Redox potential	6.1*
Root biomass	5.3**	EC	8.9*
C/N ratio	4.2*		

## Discussion

### Effect of groundwater table manipulation and biochar application on cumulative GHG emissions

#### CO_2_ emissions

This study, in agreement with prior research on agricultural peats, observed a reduction in CO_2_ emissions with increasing water table levels. Furthermore, the study highlighted the critical influence of water table depth on the extent of this reduction. (Evans et al. 2021; Koch et al. 2023; Jeewani et al. 2025). The 303.75 g CO_2_–C m^−2^ (*p* < 0.01) lower cumulative CO_2_ emission rates of mesocosms with Control + HW, clearly demonstrates that raising the water table (− 10 cm) can mitigate CO_2_ emissions from cultivated peats compared to the Control + LW (− 15 cm) showing that a 5 cm difference of water table level leads to a significant emissions reduction. This reduction in cumulative CO_2_ emission corresponds with the aerobic decomposition of the oxic peat layer. However, the cumulative CO_2_ emissions from the upper 10 cm of soil (that remains oxic) over the relatively long duration of the experiment (110 days) was still substantial (13.4–16.5 t CO_2_ ha^−1^).

Biochar application clearly led to lower rates of cumulative CO_2_ emission, compared to both the high and lower water table controls. This strongly suggests that Biochar + HW (1031.22 ± 124 g CO_2_–C m^−2^; *p* < 0.05) suppressed peat decomposition over the experimental period, by up to 615.84 g CO_2_–C m^−2^ compared to the Control + LW (1647.07 ± 148 g CO_2_−C m^−2^) treatment. The interaction between biochar and water table conditions plays a critical role in modulating GHG emissions, especially CO₂ due to its stable aromatic carbon structure, and resists microbial degradation, thereby sequestering carbon in the soil for extended periods (Lehmann et al. 2011). Evans et al. (2021) demonstrated that raising the water table in degraded peats can significantly reduce CO₂ emissions by suppressing aerobic decomposition. Under high water table conditions typical of wetlands or flooded agricultural soils, the soil becomes anaerobic, limiting microbial respiration and thus naturally reducing CO₂ emissions. In these environments, biochar further reduces CO₂ emissions by adsorbing DOC, limiting substrate availability for microbial decomposition (Singh et al. 2010). Additionally, its impact on redox potential and microbial community shifts may further suppress aerobic respiration (Cayuela et al. 2013). Conversely, in low water table (aerobic) conditions, where oxygen is more available, microbial activity and CO₂ emissions typically increase. However, biochar can still mitigate CO₂ emissions by stabilizing soil organic matter and reducing the "priming effect," wherein added carbon inputs accelerate the decomposition of native carbon stocks (Zimmerman et al. 2011). Furthermore, the porous structure of biochar improves soil aeration and water retention, buffering extreme moisture conditions and moderating microbial respiration (Lehmann and Joseph 2015). Therefore, across both anaerobic and aerobic regimes, biochar contributes to net reductions in CO₂ emissions, though the mechanisms and magnitude of this effect vary with hydrological context.

#### CH_4_ emissions

In this study, CH_4_ emissions increased by 1.5-fold for Control + HW compared to Control + LW. This is consistent with a reduction in oxygen ingress to the waterlogged soil producing anaerobic conditions, favouring methanogenic microbes (Thauer 1998; Gao et al. 2018). The addition of biochar was effective in reducing CH_4_ emissions, with Biochar+HW treatment reducing the emissions by 211.51 g CH_4_–C m^−2^ compared to Control+HW treatment, corroborating studies using biochar in mineral soils, proving mechanisms by which biochar enhances soil aeration, which can lead to increase in oxygen availability and can shift microbial processes from anaerobic (methanogenic) conditions to aerobic conditions, where methanotrophic bacteria which consume CH_4_ thrive. This shift in microbial community composition from methanogens to methanotrophs is a critical factor in reducing CH₄ emissions (Davidson et al. 2019; Sun et al. 2021).

Cumulative CH₄ emissions from the Biochar + HW and Biochar + LW treatments were nearly identical and relatively low (~ 499 mg CH₄–C m^−2^), suggesting that variations in water table depth had minimal impact on CH₄ emissions in biochar-amended peat soils. This is likely due to improved redox conditions and oxygen availability in the upper soil layers. This oxygen inhibits methanogenesis by suppressing anaerobic microbes responsible for CH₄ production and supports the activity of methanotrophs, which oxidize CH₄ as it ascends through the soil. However, even under these conditions, CH₄ can bypass oxidation if its rate of production exceeds the methanotrophic capacity. This may occur due to rapid diffusive transport, limited CH₄ solubility in pore water, or spatial heterogeneity in oxygen availability, allowing some CH₄ to escape into the atmosphere before being oxidized (Mohanty et al. 2014; Ma et al. 2022). Furthermore, CH₄ suppression was maintained until water table decline exceeded 20 cm, consistent with previous findings that a well-managed high-water level can effectively reduce emissions (Evans et al. 2024). Interestingly, under low water table conditions (LW), biochar alone appeared to enhance CH₄ emissions compared to Control + LW, suggesting that under more aerated conditions, biochar may stimulate methanogenesis by providing labile carbon and enhancing microbial electron transfer, while also potentially inhibiting CH₄ oxidation by altering oxygen diffusion, microbial community structure, or CH₄ availability to methanotrophs (Spokas et al. 2009; Cayuela et al. 2013; Lu et al. 2022).

The observed CO_2_ equivalent GHG emissions from CH₄ (0.05–0.19 t CO₂ − eq ha⁻^1^ yr⁻^1^) were significantly lower than those from CO₂, aligning with field and mesocosm data from the same site, where CH₄ emissions typically average around 0.04 t CO₂ − eq ha⁻^1^ yr⁻^1^ (Musarika et al. 2017; Taft et al. 2018). In UK lowland peats, CH₄ emissions are generally negligible when water tables are maintained more than 20 cm below the surface (Evans et al. 2017, 2021; Matysek et al. 2019). Matysek et al. (2019) also found very low CH₄ fluxes in celery-grown mesocosms with a − 50 cm water table. These findings indicate that significantly elevated CH₄ emissions are unlikely under moderately raised, but still subsurface, water tables aimed at mitigating CO₂ and N₂O losses from cultivated peats.

#### N_2_O emissions

Raising the water table reduced N_2_O emissions in comparison to the low water table control treatment. We ascribe this to a reduced microbial activity and lower availability of NO_3_^−^ and available carbon to drive denitrification (Liu et al. 2016; Liimatainen et al. 2018). This is consistent with field studies on agricultural peat that have shown increased N_2_O emissions after soil drainage and that this is due to nitrification of the NH_4_^+^ generated during the high rates of peat mineralisation under aerobic conditions (Liimatainen et al. 2018; Taghizadeh–Toosi et al. 2019). Previous studies have also recorded reduced N_2_O emissions when the groundwater level is raised, but elevated emissions when the groundwater level is lowered (van Beek et al. 2011; Taft et al. 2018). This is consistent with findings from Freeman et al. (1996) who also found N_2_O emission to be inversely correlated with the depth of water table. The highest rate of N₂O emission in our study was observed in the Control + LW treatment after 110 days (589 ± 88 mg N₂O m⁻^2^), which was of a lower magnitude and comparable to emissions reported in previous studies on arable peatlands (Freeman et al. 1996, 2022; Taft et al. 2018). In contrast, the cumulative N₂O–N emissions from the Control + HW, Biochar + HW and Biochar + LW treatments were lower. These responses to wet peat soils are typical, with N₂O emissions generally being limited by soil moisture and soluble nitrogen availability (Liu et al. 2022). Additionally, low N₂O emissions were observed during lettuce growth across all treatments, likely due to the crop uptake of soil nitrogen, which reduced the availability of substrates for nitrification and denitrification (Matysek et al. 2022).

Based on 100-year global warming potentials for CH_4_ and N_2_O, overall GHG emissions were highest for the Control + LW (16.3 t CO_2_eq ha^−1^ yr^−1^), as a result of enhancing oxygen and mineral nitrogen availability (Klemedtsson et al. 2005; Pärn et al. 2018). In the longer term, however, the radiative forcing benefits of conserving peat C stocks via re-wetting can be expected to outweigh the costs of higher CH_4_ emissions, due to the shorter atmospheric lifetime of CH_4_ (Günther et al. 2020).

### Biochar and water table level mediated microbial community shift and its relation to GHG emission

The structure and functional dynamics of the soil microbiome are strongly modulated by the availability of soil moisture and the presence of biochar amendments. It was reported the microbial processes are directly impacted by factors such as water table levels, vegetation productivity, soil temperature, the availability of readily decomposable organic matter (labile carbon), and the presence of oxidizing agents (e.g. Fe(III) oxides and SO_4_^2−^) in the peat (Dean et al. 2018). We observed that water table level exhibited a significant effect on both bacterial and fungal abundance (Fig. 3a, b), which suggests that the soil microbial community is sensitive to changes in soil moisture and oxygen availability (Churchill et al. 2015). Bacterial composition has been shown to be affected by short-term water table drawdown, whose ecological niche is dependent on the position of the water table (Jaatinen et al. 2005).

Actinobacteria abundance was highest in Control + LW (Fig. 4a). Previous studies have shown that Actinobacteria can contribute to SOC decomposition through their mycelial growth, which enhances access to organic matter (Luo et al. 2017; Fu et al. 2022). This increased SOC decomposition, as evidenced by higher CO₂ emissions, may result from either direct priming effects by Actinobacteria or from a combination of priming and rapid microbial biomass turnover (Luo et al. 2011). LW treatments further have been shown to increase the abundance of the genus *Alicyclobacillus*, which have the ability to assimilate various carbon sources (fatty acids) and to oxidize mineral associated organic complexes and sulfuric compounds (Jiang et al. 2008). Overall, our studies alongside with previous studies suggest that raising water table of peat may decrease bacterial diversity and alter the net functioning of bacterial communities (Fig. 3c, d). Biochar addition has been shown to shift the relative abundance of *Occallatibacter* (affiliated to Acidobacteria) and *Pseudomonas* (affiliated to Proteobacteria). The genus *Occallatibacter* is adapted to acidic environments; hence if biochar additions raise soil pH, *Occallatibacter* abundance decreases*,* which may lead to a reduction in the efficiency of SOC decomposition (Lladó et al. 2016). The potential explanation for the increase in Proteobacteria abundance in our study is that biochar increased soil labile C, soil pH and improved aeration and soil stucture, providing a favourable microenvironment. Proteobacteria thrive in neutral to slightly alkaline conditions, so this shift could have favored their growth over other microbial groups (Lladó et al. 2016). The observed increase in Proteobacteria abundance following biochar application could be attributed to several factors. Firstly, the alkaline nature of the biochar likely increased the soil pH in our acidic experimental plots, creating a more favorable environment for many Proteobacteria species known to thrive in neutral to slightly alkaline conditions. It was reported that the most biochar produced from woody feedstocks at high pyrolysis temperatures (above 500 °C) is alkaline due to the presence of base cations such as calcium, potassium, and magnesium (Lehmann et al. 2011). Secondly, the initial weathering of biochar might have released labile carbon compounds, providing a readily available carbon source that metabolically versatile Proteobacteria could quickly utilize, leading to their proliferation (Taghizadeh−Toosi et al. 2012). Furthermore, the improved soil structure and aeration resulting from biochar amendment could have enhanced oxygen availability, benefiting aerobic Proteobacteria.

Ascomycota (which are *K-*strategist) was the dominant phylum of fungi in all treatments (73–80%), especially highest in Control + LW (80% relative abundance). It was reported that Ascomycota is a most frequently isolated from peat (63%) and a functionally diverse phylum known to have a high metabolic diversity and substrate versatility and is therefore better able to adapt to oligotrophic conditions. Moreover, the ability of Ascomycota to produce secondary metabolites, and their mycelial growth habit make it possible to explore the carbon sources, water and other nutrients. The abundance of the genera *Gibellulopsis* and *Chaetomium* was significantly increased in the biochar-amended soils (Bamminger et al. 2014; Yao et al. 2017). *Gibellulopsis* is reportedly associated with carbon and it plays a key role in the breakdown of hemicellulose, cellulose and lignin (López et al. 2021). Furthermore, biochar significantly increased the relative abundance of *Chaetomium* (Fig. 3d), aligning the increased abundance of potential biocontrol fungi in biochar-amended treatments. Biochar-mediated changes of the rhizosphere fungal community, especially the enrichment of biocontrol fungi, are closely related to the suppression of soil-borne diseases (Yao et al. 2017; Wang et al. 2020). The abundance of potential biocontrol fungi in the rhizosphere soil, coupled with the potential for improved crop quality and biomass, suggests that biochar could offer a sustainable and economically viable solution for managing soilborne diseases.

Greenhouse gas emissions in peat, and their response to biochar amendments are both complex and critical to understanding the broader implications for climate mitigation. Biochar influences microbial community composition, abundance, and diversity (Fig. 4), which in turn affects the production and consumption of CO₂, CH₄, and N₂O. By altering soil pH, nutrient availability, aeration, and moisture retention, biochar indirectly shapes the microhabitats that support specific microbial guilds. For instance, biochar can enhance the abundance of nitrifiers and denitrifiers, but its impact on N₂O emissions depends on how it influences the balance between complete and incomplete denitrification (Anderson et al. 2014). Additionally, the porous structure of biochar provides refuge and colonization surfaces for methanotrophs, potentially lowering CH_4_ emission. This physical protection may also reduce microbial predation and desiccation, leading to increased enzymatic activity involved in nutrient cycling (Lehmann et al. 2011). Importantly, biochar has been shown to suppress methanogenic archaea in anaerobic zones while promoting methanotrophic bacteria (genera related to Proteobacteria and Verrucomicrobiota) in oxic microsites, which collectively contribute to lower net CH₄ emissions (Cayuela et al. 2013). These microbial shifts are not merely additive but involve complex interactions between microbial trophic levels and functional groups, modulated by changes in redox potential, electron donor/acceptor availability, and competition for substrates. Thus, biochar not only alters microbial processes individually but also influences the network-level interactions that ultimately drive GHG fluxes in soils.

### Relationships and contributions of soil and plant variables on lettuce biomass and microbial community

Total biomass of lettuce across both crops was increased in biochar treatments compared to controls and significantly higher in Biochar + LW than in Biochar + HW (Fig. 4a), which is consistent with findings on celery biomass (Lin et al. 2023). Biochar application offers numerous benefits beyond C addition and GHG emission reduction. It enhances soil health by improving soil pH, moisture retention, physical structure, nutrient availability, and biological activity (Fig. 2 and Table 1). These improvements ultimately contribute to enhanced plant growth and resilience under various stress conditions (Sohi et al. 2010; Meng et al. 2019).

A higher root biomass was observed in the Biochar + LW treatment compared to Biochar + HW, indicating that elevating the groundwater table up to 10 cm from the soil surface positively affects root development (Armstrong and Drew 2002). In this study, lettuce root growth was restricted to the area above the groundwater table. Additionally, elevated groundwater levels may limit above- and below-ground biomass due to nutrient constraints. These constraints arise from (i) restricted access to deeper nutrient pools by plants and their symbiotic partners (Oomes et al. 1996) and (ii) reduced nutrient release from peat and biochar mineralization.

In this study, multiple soil and plant edaphic factors were found to significantly influence the composition of soil microbial communities. For bacteria, pH (8.2%), DOC (7.9%), soil moisture (7.1%), and NO₃⁻ (6.4%) emerged as key drivers. These variables are well-established determinants of bacterial diversity and activity, as they directly affect microbial metabolism, nutrient availability, and environmental tolerances. Similarly, fungal communities were primarily influenced by NH₄⁺ (10.1%), TC (8.9%), soil moisture (8.5%), and EC (8.9%), suggesting that nitrogen availability and organic matter content play dominant roles in shaping fungal assemblages. In addition to these abiotic factors, root biomass (5.3%) significantly contributed to bacterial community variation, reflecting the strong influence of rhizosphere processes. Root-derived exudates and root turnover create microhabitats that facilitate microbial colonization and selective enrichment of root-associated taxa (Philippot et al. 2013). Thus, plant–microbe interactions are an essential biotic component in structuring microbial communities.

Soil moisture content is a main determinant factor contributing fungal (8.5%) and bacterial community (7.1%) underscoring the crucial role of water in supporting microbial community and diversity. Some fungi such as phylum Ascomycota are more tolerant to drier conditions, while others thrive in moist environments. Moisture can also influence the oxygen availability, which affects the distribution of aerobic and anaerobic fungi (Xiong et al. 2022). The higher significance (*p* < 0.01) suggests a strong link between soil moisture and fungal community structure (Table 2).

pH is a dominant factor shaping bacterial communities, influencing microbial enzyme activity, nutrient availability, and species survival. Bacteria exhibit a wide range of pH and shifts in pH can selectively favor certain bacterial taxa over others, leading to changes in community composition (Fierer and Jackson 2006). Bacteria often have narrower pH tolerance than fungi. Both ammonium and nitrate concentrations appear to be strong drivers of both bacterial and fungal community composition, highlighting the importance of nitrogen cycling in shaping soil microbial communities. Fungi are more sensitive to NH₄⁺ and total carbon, possibly reflecting their role in nitrogen cycling and decomposition. Its concentration can directly influence bacterial composition, and the relative abundance of different bacterial groups involved in nitrogen cycling (e.g., nitrifiers, denitrifiers) and general metabolism (Kirkby 2012).

Root biomass contributed 5.3% to the variation in bacterial community composition, highlighting its role in shaping rhizosphere microbial dynamics. This influence is largely attributed to root exudation, a process through which plants release a diverse array of organic compounds such as sugars, amino acids, and secondary metabolites into the surrounding soil. These exudates create a nutrient-rich microenvironment in the rhizosphere that selectively stimulates the growth and activity of specific bacterial taxa, thereby contributing to a distinct microbial community structure. Greater root biomass generally corresponds to increased rhizodeposition, which in turn enhances microbial recruitment, colonization, and functional interactions within the rhizosphere. As such, the structure and function of the root-associated bacterial community are closely linked to plant root development (Philippot et al. 2013).

**Fig. 5:**
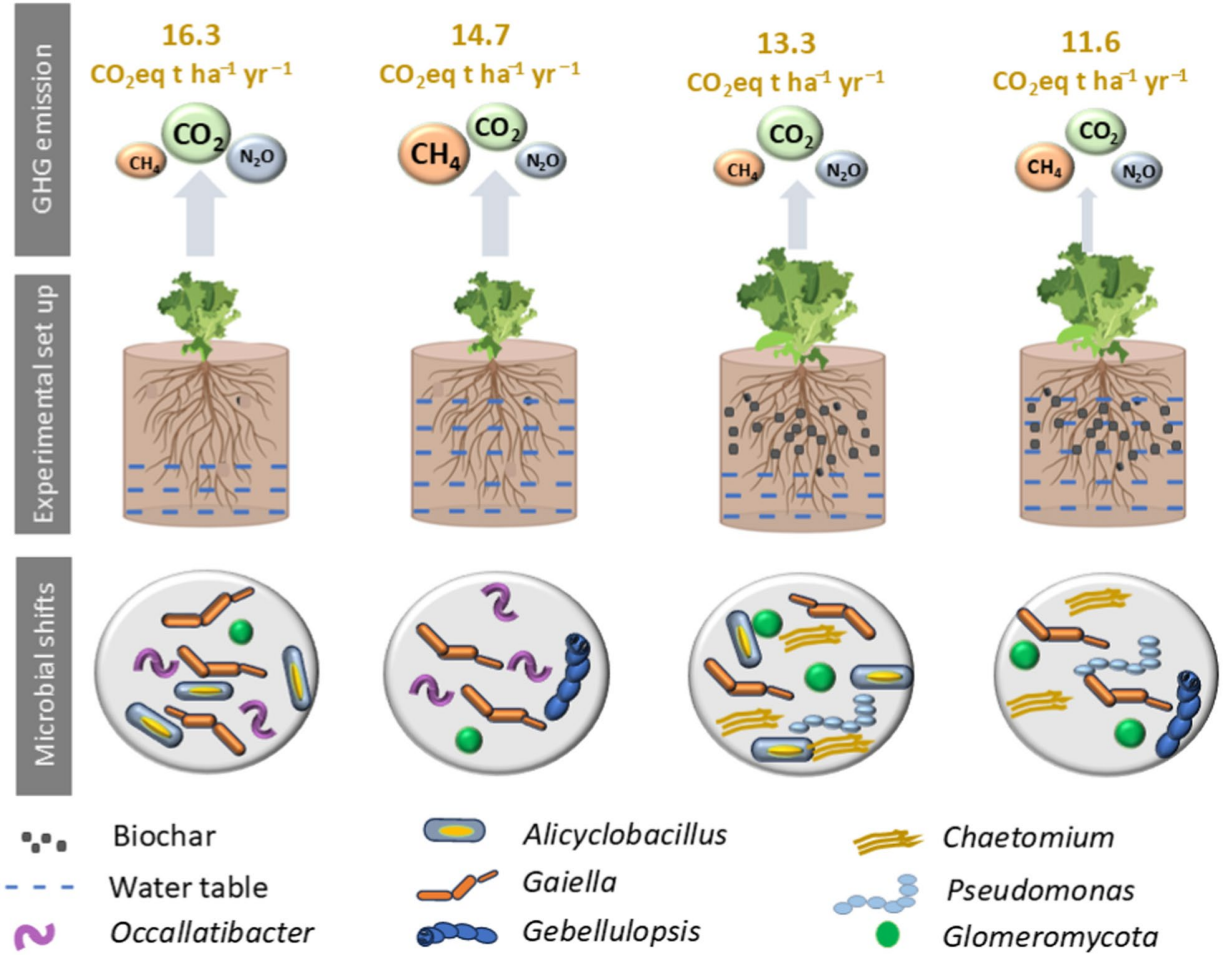
Conceptual diagram of the mesocosm set up, estimated greenhouse gas balance and core microbial taxa for peat mesocosms with and without biochar amendmends and water table management [− 10 cm (high water table), and − 15 cm (low water table)] from the soil surface

Taken together, this study focuses on the short-term effects of biochar over two lettuce growing seasons, and it is important to consider the potential long-term implications of biochar application on both crop productivity and GHG emissions (Fig. 5). Biochar is characterized by its chemical stability and slow decomposition rate, meaning its effects can persist in soil for years to decades (Lehmann and Rondon 2006). Over the long term, biochar can continue to improve soil structure, enhance nutrient retention, and buffer pH, which cumulatively support sustained or even increased crop yields (Jeffery et al. 2011). Furthermore, repeated or residual effects of biochar may promote the development of more resilient soil microbial communities, contributing to enhanced nutrient cycling and improved soil health. In terms of GHG emissions, the long-term C stabilization in the form of stable aromatic compounds reduces net CO₂ emissions from the soil, while ongoing reductions in N₂O and CH₄ emissions are also possible, particularly when biochar improves soil aeration and nitrogen use efficiency (Spokas et al. 2012; Schmidt et al. 2014).

## Conclusions

The combination of raised groundwater levels and biochar amendments substantially reduced overall GHG emissions, especially of CO_2_. Biochar application significantly enhanced lettuce biomass, even in the higher water level treatment. Ultimately, this may also lead to increased C addition to soil via a greater return of crop residues. Our findings have major practical and economic implications, suggesting that it may be possible to farm peats productively for food crops whilst retaining or even enhancing carbon stores, and avoiding offsetting emissions of non-CO_2_ GHGs. Positive biomass responses to biochar application would greatly enhance the economic case for biochar as a climate mitigation measure for agricultural peats by providing a direct economic return to farmers. Although our results require further testing and verification at a field scale over multiple cropping cycles, they are among the first to suggest that it may be possible to break the trade-off between food production and climate change mitigation in agricultural peats.

## Supplementary Information

Supplementary material 1.

## Data Availability

Data will be made available on request.

## References

[CR500] Anderson MJ (2003) DISTLM forward: a FORTRAN computer program to calculate a distance-based multivariate analysis for a linear model using forward selection. Department of Statistics, University of Auckland, New Zealand, pp 10

[CR1] Anderson MJ, Legendre P (1999) An empirical comparison of permutation methods for tests of partial regression coefficients in a linear model. J Stat Comput Simul 62:271–303

[CR2] Anderson CR, Hamonts K, Clough TJ, Condron LM (2014) Biochar does not affect soil N−transformations or microbial community structure under ruminant urine patches but does alter relative proportions of nitrogen cycling bacteria. Agr Ecosyst Environ 191:63–72

[CR3] Armstrong W, Drew MC (2002) Root growth and metabolism under oxygen deficiency, plant roots. CRC Press, Boca Raton, pp 1139–1187

[CR4] Bamminger C, Zaiser N, Zinsser P, Lamers M, Kammann C, Marhan S (2014) Effects of biochar, earthworms, and litter addition on soil microbial activity and abundance in a temperate agricultural soil. Biol Fertil Soils 50:1189–1200

[CR5] Biederman LA, Harpole WS (2013) Biochar and its effects on plant productivity and nutrient cycling: a meta−analysis. GCB Bioenergy 5:202–214

[CR6] Cayuela ML, Sanchez-Monedero MA, Roig A, Hanley K, Enders A, Lehmann J (2013) Biochar and denitrification in soils: when, how much and why does biochar reduce N2O emissions? Sci Rep 3:1732 23615819 10.1038/srep01732 PMC3635057

[CR7] Churchill AC, Turetsky MR, McGuire AD, Hollingsworth TN (2015) Response of plant community structure and primary productivity to experimental drought and flooding in an Alaskan fen. Can J for Res 45:185–193

[CR8] Davidson SJ, Van Beest C, Petrone R, Strack M (2019) Wildfire overrides hydrological controls on boreal peatland methane emissions. Biogeosciences 16:2651–2660

[CR9] Dawson Q, Kechavarzi C, Leeds-Harrison PB, Burton RGO (2010) Subsidence and degradation of agricultural peatlands in the Fenlands of Norfolk, UK. Geoderma 154:181–187

[CR10] Dean JF, Middelburg JJ, Röckmann T, Aerts R, Blauw LG, Egger M, Jetten MSM, de Jong AEE, Meisel OH, Rasigraf O (2018) Methane feedbacks to the global climate system in a warmer world. Rev Geophys 56:207–250

[CR501] Downie A, Crosky A, Munroe P (2009) Physical properties of biochar. In: Lehmann J, Joseph S (eds) Biochar for Environmental Management: Science and Technology, Earthscan, London, pp 13–32

[CR11] Evans CD, Peacock M, Baird AJ, Artz RRE, Burden A, Callaghan N, Chapman PJ, Cooper HM, Coyle M, Craig E (2021) Overriding water table control on managed peatland greenhouse gas emissions. Nature 593:548–552 33882562 10.1038/s41586-021-03523-1

[CR12] Evans CD, Rowe RL, Freeman BWJ, Rhymes JM, Cumming A, Lloyd IL, Morton D, Williamson JL, Morrison R (2024) Biomethane produced from maize grown on peat emits more CO2 than natural gas. Nat Clim Chang 14:1030–1032

[CR13] Evans C, Artz R, Moxley J, Smyth M-A, Taylor E, Archer E, Burden A, Williamson J, Donnelly D, Thomson A (2017) Implementation of an emissions inventory for UK peatlands. Centre for Ecology and Hydrology

[CR14] Evans CD, Morrison R, Cumming A, Bodo A, Burden A, Callaghan N, Clilverd H, Cooper H, Cowan N, Crabtree D (2023) Defra Lowland Peat 2: Managing agricultural systems on lowland peat for decreased greenhouse gas emissions whilst maintaining agricultural productivity. Report to Defra for Project SP1218

[CR901] Fierer N, Jackson RB (2006) The diversity and biogeography of soil bacterial communities. Proc National Acad Sci 103:626–631 10.1073/pnas.0507535103 PMC1334650 16407148

[CR15] Freeman C, Liska G, Ostle NJ, Lock MA, Reynolds B, Hudson J (1996) Microbial activity and enzymic decomposition processes following peatland water table drawdown. Plant Soil 180:121–127

[CR16] Freeman BWJ, Evans CD, Musarika S, Morrison R, Newman TR, Page SE, Wiggs GFS, Bell NGA, Styles D, Wen Y, Chadwick DR, Jones DL (2022) Responsible agriculture must adapt to the wetland character of mid−latitude peatlands. Glob Change Biol 28:3795–3811 10.1111/gcb.16152 PMC9314663 35243734

[CR17] Fu Y, Luo Y, Auwal M, Singh BP, Van Zwieten L, Xu J (2022) Biochar accelerates soil organic carbon mineralization via rhizodeposit−activated actinobacteria. Biol Fertil Soils 58:565–577

[CR18] Gao J, Hou H, Zhai Y, Woodward A, Vardoulakis S, Kovats S, Wilkinson P, Li L, Song X, Xu L, Meng B, Liu X, Wang J, Zhao J, Liu Q (2018) Greenhouse gas emissions reduction in different economic sectors: Mitigation measures, health co−benefits, knowledge gaps, and policy implications. Environ Pollut 240:683–698 29775945 10.1016/j.envpol.2018.05.011

[CR19] Günther A, Barthelmes A, Huth V, Joosten H, Jurasinski G, Koebsch F, Couwenberg J (2020) Prompt rewetting of drained peatlands reduces climate warming despite methane emissions. Nat Commun 11:1644 32242055 10.1038/s41467-020-15499-z PMC7118086

[CR20] Hou J, Pugazhendhi A, Sindhu R, Vinayak V, Thanh NC, Brindhadevi K, Lan Chi NT, Yuan D (2022) An assessment of biochar as a potential amendment to enhance plant nutrient uptake. Environ Res 214:113909 35850292 10.1016/j.envres.2022.113909

[CR502] IPCC, Gutiérrez JM, Jones RG, Narisma GT (2024) IPCC Sixth Assessment Report (AR6) Technical Summary. NERC EDS Centre for Environmental Data Analysis

[CR21] Jaatinen K, Tuittila ES, Laine J, Yrjälä K, Fritze H (2005) Methane−oxidizing bacteria in a Finnish raised mire complex: effects of site fertility and drainage. Microb Ecol 50:429–439 16283115 10.1007/s00248-005-9219-x

[CR22] Jeewani PH, Brown RW, Rhymes JM, McNamara NP, Chadwick DR, Jones DL, Evans CD (2025) Greenhouse gas removal in agricultural peatland via raised water levels and soil amendment. Biochar 7:39 39991092 10.1007/s42773-024-00422-2 PMC11845426

[CR503] Jeffery S, Verheijen FG, van der Velde M, Bastos AC (2011) A quantitative review of the effects of biochar application to soils on crop productivity using meta-analysis. Agric Ecosyst Environ 144(1):175–187

[CR23] Jiang C-Y, Liu Y, Liu Y-Y, You X-Y, Guo X, Liu S-J (2008) Alicyclobacillus ferrooxydans sp. nov., a ferrous−oxidizing bacterium from solfataric soil. Int J Syst Evolut Microbiol 58:2898–2903 10.1099/ijs.0.2008/000562-0 19060079

[CR24] Joosten H, Sirin A, Couwenberg J, Laine J, Smith P (2016) The role of peatlands in climate regulation. Cambridge University Press, Cambridge

[CR505] Keiluweit M, Nico PS, Johnson MG, Kleber M (2010) Dynamic molecular structure of plant biomass-derived black carbon (Biochar). Environ Sci Technol 44:1247–1253 10.1021/es9031419 20099810

[CR601] Kirkby E (2012) Introduction, definition and classification of nutrients. In: Marschner H (ed) Marschner's mineral nutrition of higher plants, 3rd edn. Academic, Amsterdam, pp 3–5

[CR25] Klemedtsson L, Von Arnold K, Weslien P, Gundersen P (2005) Soil CN ratio as a scalar parameter to predict nitrous oxide emissions. Glob Change Biol 11:1142–1147

[CR26] Koch J, Elsgaard L, Greve MH, Gyldenkærne S, Hermansen C, Levin G, Wu S, Stisen S (2023) Water−table−driven greenhouse gas emission estimates guide peatland restoration at national scale. Biogeosciences 20:2387–2403

[CR506] Lehmann J, Joseph S (2015) Biochar for environmental management: an introduction. In: Biochar for environmental management. Routledge, pp 1–13

[CR507] Lehmann J, Rondon M (2006) Bio-char soil management on highly weathered soils in the humid tropics. Biol Approaches Sust Soil Syst 113(517):e530

[CR27] Lehmann J, Rillig MC, Thies J, Masiello CA, Hockaday WC, Crowley D (2011) Biochar effects on soil biota–a review. Soil Biol Biochem 43:1812–1836

[CR28] Leifeld J, Menichetti L (2018) The underappreciated potential of peatlands in global climate change mitigation strategies. Nat Commun 9:1071 29540695 10.1038/s41467-018-03406-6 PMC5851997

[CR29] Leifeld J, Müller M, Fuhrer J (2011) Peatland subsidence and carbon loss from drained temperate fens. Soil Use Manag 27:170–176

[CR30] Leifeld J, Wüst-Galley C, Page S (2019) Intact and managed peatland soils as a source and sink of GHGs from 1850 to 2100. Nat Clim Change 9:945–947

[CR31] Liimatainen M, Voigt C, Martikainen PJ, Hytönen J, Regina K, Óskarsson H, Maljanen M (2018) Factors controlling nitrous oxide emissions from managed northern peat soils with low carbon to nitrogen ratio. Soil Biol Biochem 122:186–195

[CR32] Lin C-C, Liu Y-T, Chang P-H, Hsieh Y-C, Tzou Y-M (2023) Inhibition of continuous cropping obstacle of celery by chemically modified biochar: an efficient approach to decrease bioavailability of phenolic allelochemicals. J Environ Manag 348:119316 10.1016/j.jenvman.2023.119316 37862893

[CR33] Liu R, Hu H, Suter H, Hayden HL, He J, Mele P, Chen D (2016) Nitrification is a primary driver of nitrous oxide production in laboratory microcosms from different land−use soils. Front Microbiol 7:1373 27667985 10.3389/fmicb.2016.01373 PMC5016788

[CR34] Liu S, He F, Kuzyakov Y, Xiao H, Hoang DTT, Pu S, Razavi BS (2022) Nutrients in the rhizosphere: a meta−analysis of content, availability, and influencing factors. Sci Total Environ 826:153908 35183641 10.1016/j.scitotenv.2022.153908

[CR35] Lladó S, Žifčáková L, Větrovský T, Eichlerová I, Baldrian P (2016) Functional screening of abundant bacteria from acidic forest soil indicates the metabolic potential of Acidobacteria subdivision 1 for polysaccharide decomposition. Biol Fertil Soils 52:251–260

[CR36] López MJ, Jurado MM, López-González JA, Estrella-González MJ, Martínez-Gallardo MR, Toribio A, Suárez-Estrella F (2021) Characterization of thermophilic lignocellulolytic microorganisms in composting. Front Microbiol 12:697480 34456885 10.3389/fmicb.2021.697480 PMC8385673

[CR37] Lu Y, Liu Q, Fu L, Hu Y, Zhong L, Zhang S, Liu Q, Xie Q (2022) The effect of modified biochar on methane emission and succession of methanogenic archaeal community in paddy soil. Chemosphere 304:135288 35691388 10.1016/j.chemosphere.2022.135288

[CR38] Luo Y, Durenkamp M, De Nobili M, Lin Q, Brookes PC (2011) Short term soil priming effects and the mineralisation of biochar following its incorporation to soils of different pH. Soil Biol Biochem 43:2304–2314

[CR39] Luo L, Meng H, Gu J-D (2017) Microbial extracellular enzymes in biogeochemical cycling of ecosystems. J Environ Manag 197:539–549 10.1016/j.jenvman.2017.04.023 28419976

[CR40] Lyu H, Zhang H, Chu M, Zhang C, Tang J, Chang SX, Mašek O, Ok YS (2022) Biochar affects greenhouse gas emissions in various environments: a critical review. Land Degrad Dev 33:3327–3342

[CR41] Ma L, Zhu G, Chen B, Zhang K, Niu S, Wang J, Ciais P, Zuo H (2022) A globally robust relationship between water table decline, subsidence rate, and carbon release from peatlands. Commu Earth Environ 3:254

[CR42] Ma X, Li S, Pan R, Wang Z, Li J, Zhang X, Azeem M, Yao Y, Xu Z, Pan J, Zhang Z, Li R (2023) Effect of biochar on the mitigation of organic volatile fatty acid emission during aerobic biostabilization of biosolids and the underlying mechanism. J Clean Prod 390:136213

[CR43] Matysek M, Leake J, Banwart S, Johnson I, Page S, Kaduk J, Smalley A, Cumming A, Zona D (2019) Impact of fertiliser, water table, and warming on celery yield and CO2 and CH4 emissions from fenland agricultural peat. Sci Total Environ 667:179–190 30826678 10.1016/j.scitotenv.2019.02.360

[CR44] Matysek M, Leake J, Banwart S, Johnson I, Page S, Kaduk J, Smalley A, Cumming A, Zona D (2022) Optimizing fen peatland water-table depth for romaine lettuce growth to reduce peat wastage under future climate warming. Soil Use Manag 38:341–354

[CR510] McNicol G, Fluet‐Chouinard E, Ouyang Z, Knox S, Zhang Z, Aalto T, Bansal S, Chang KY, Chen M, Delwiche K (2023). Upscaling wetland methane emissions from the FLUXNET‐CH4 eddy covariance network (UpCH4 v1. 0): Model development, network assessment, and budget comparison. AGU Adv 4:e2023AV000956

[CR45] Meng L, Sun T, Li M, Saleem M, Zhang Q, Wang C (2019) Soil−applied biochar increases microbial diversity and wheat plant performance under herbicide fomesafen stress. Ecotoxicol Environ Saf 171:75–83 30597319 10.1016/j.ecoenv.2018.12.065

[CR511] Miranda KM, Espey MG, Wink DA (2001) A rapid, simple spectrophotometric method for simultaneous detection of nitrate and nitrite. Nitric Oxide 5:62–71 10.1006/niox.2000.0319 11178938

[CR46] Mohanty SR, Kollah B, Sharma VK, Singh AB, Singh M, Rao AS (2014) Methane oxidation and methane driven redox process during sequential reduction of a flooded soil ecosystem. Ann Microbiol 64:65–74

[CR513] Mulvaney RL (1996) Nitrogen—inorganic forms. Methods Soil Anal Part 3 Chem Methods 5:1123–1184

[CR47] Musarika S, Atherton CE, Gomersall T, Wells MJ, Kaduk J, Cumming AMJ, Page SE, Oechel WC, Zona D (2017) Effect of water table management and elevated CO2 on radish productivity and on CH4 and CO2 fluxes from peatlands converted to agriculture. Sci Total Environ 584:665–672 28153403 10.1016/j.scitotenv.2017.01.094

[CR520] Oomes MJM, Olff H, Altena HJ (1996) Effects of vegetation management and raising the water table on nutrient dynamics and vegetation change in a wet grassland. J Appl Ecol 576–588

[CR48] Page S, Baird A, Cumming A, High KE, Kaduk J, Evans C (2020) An assessment of the societal impacts of water level management on lowland peatlands in England and Wales: Report to Defra for Project SP1218: Managing agricultural systems on lowland peat for decreased greenhouse gas emissions whilst maintaining agricultural productivity

[CR49] Palansooriya KN, Ok YS, Awad YM, Lee SS, Sung J-K, Koutsospyros A, Moon DH (2019) Impacts of biochar application on upland agriculture: a review. J Environ Manag 234:52–64 10.1016/j.jenvman.2018.12.085 30616189

[CR50] Pärn J, Verhoeven JTA, Butterbach-Bahl K, Dise NB, Ullah S, Aasa A, Egorov S, Espenberg M, Järveoja J, Jauhiainen J (2018) Nitrogen-rich organic soils under warm well−drained conditions are global nitrous oxide emission hotspots. Nat Commun 9:1–8 29555906 10.1038/s41467-018-03540-1 PMC5859301

[CR550] Philippot L, Raaijmakers JM, Lemanceau P, Van Der Putten WH (2013) Going back to the roots: the microbial ecology of the rhizosphere. Nat Rev Microbiol 11(11):789–799 10.1038/nrmicro3109 24056930

[CR51] Prananto JA, Minasny B, Comeau LP, Rudiyanto R, Grace P (2020) Drainage increases CO2 and N2O emissions from tropical peat soils. Glob Change Biol 26:4583–4600 10.1111/gcb.15147 32391633

[CR600] Schmidt KB, Treu T, Trenti M, Bradley LD, Kelly BC, Oesch PA, Holwerda BW, Shull JM, Stiavelli M (2014) The luminosity function at z∼ 8 from 97 Y-band dropouts: Inferences about reionization. Astrophys J 786(1):57

[CR52] Singh BK, Bardgett RD, Smith P, Reay DS (2010) Microorganisms and climate change: terrestrial feedbacks and mitigation options. Nat Rev Microbiol 8:779–790 20948551 10.1038/nrmicro2439

[CR53] Sohi SP, Krull E, Lopez-Capel E, Bol R (2010) A review of biochar and its use and function in soil. Adv Agron 105:47–82

[CR54] Spokas KA, Koskinen WC, Baker JM, Reicosky DC (2009) Impacts of woodchip biochar additions on greenhouse gas production and sorption/degradation of two herbicides in a Minnesota soil. Chemosphere 77:574–581 19647284 10.1016/j.chemosphere.2009.06.053

[CR800] Spokas KA, Cantrell KB, Novak JM, Archer DW, Ippolito JA, Collins HP, Boateng AA, Lima IM, Lamb MC, McAloon AJ, Lentz RD (2012) Biochar: a synthesis of its agronomic impact beyond carbon sequestration. J Environ Qual 41(4):973–989. 10.2134/jeq2011.0069 22751040

[CR55] Sun T, Guzman JJL, Seward JD, Enders A, Yavitt JB, Lehmann J, Angenent LT (2021) Suppressing peatland methane production by electron snorkeling through pyrogenic carbon in controlled laboratory incubations. Nat Commun 12:4119 34226558 10.1038/s41467-021-24350-y PMC8257765

[CR56] Taft HE, Cross PA, Jones DL (2018) Efficacy of mitigation measures for reducing greenhouse gas emissions from intensively cultivated peatlands. Soil Biol Biochem 127:10–21

[CR57] Taghizadeh-Toosi A, Clough TJ, Sherlock RR, Condron LM (2012) Biochar adsorbed ammonia is bioavailable. Plant Soil 350:57–69

[CR58] Taghizadeh-Toosi A, Elsgaard L, Clough TJ, Labouriau R, Ernstsen V, Petersen SO (2019) Regulation of N 2 O emissions from acid organic soil drained for agriculture. Biogeosciences 16:4555–4575

[CR59] Thauer RK (1998) Biochemistry of methanogenesis: a tribute to Marjory Stephenson. 1998 marjory stephenson prize lecture. Microbiology 144:2377–2406 9782487 10.1099/00221287-144-9-2377

[CR60] Tiemeyer B, Albiac Borraz E, Augustin J, Bechtold M, Beetz S, Beyer C, Drösler M, Ebli M, Eickenscheidt T, Fiedler S, Förster C, Freibauer A, Giebels M, Glatzel S, Heinichen J, Hoffmann M, Höper H, Jurasinski G, Leiber-Sauheitl K, Peichl-Brak M, Roßkopf N, Sommer M, Zeitz J (2016) High emissions of greenhouse gases from grasslands on peat and other organic soils. Glob Change Biol 22:4134–4149 10.1111/gcb.13303 27029402

[CR61] van Beek CL, Pleijter M, Kuikman PJ (2011) Nitrous oxide emissions from fertilized and unfertilized grasslands on peat soil. Nutr Cycl Agroecosyst 89:453–461

[CR62] Wang G, Ma Y, Chenia HY, Govinden R, Luo J, Ren G (2020) Biochar−mediated control of phytophthora blight of pepper is closely related to the improvement of the rhizosphere fungal community. Front Microbiol 11:1427 32733402 10.3389/fmicb.2020.01427 PMC7360685

[CR63] Wang Y, Calanca P, Leifeld J (2024) Sources of nitrous oxide emissions from agriculturally managed peatlands. Glob Change Biol 30:e17144 10.1111/gcb.17144 38273517

[CR64] Wen Y, Zang H, Freeman B, Ma Q, Chadwick DR, Jones DL (2019) Rye cover crop incorporation and high watertable mitigate greenhouse gas emissions in cultivated peatland. Land Degrad Dev 30:1928–1938

[CR65] Xiong B-J, Kleinsteuber S, Sträuber H, Dusny C, Harms H, Wick LY (2022) Impact of fungal hyphae on growth and dispersal of obligate anaerobic bacteria in aerated habitats. Mbio 13:e00769-00722 35638736 10.1128/mbio.00769-22 PMC9239063

[CR66] Yao Q, Liu J, Yu Z, Li Y, Jin J, Liu X, Wang G (2017) Three years of biochar amendment alters soil physiochemical properties and fungal community composition in a black soil of northeast China. Soil Biol Biochem 110:56–67

[CR67] Yin J, Zhao L, Xu X, Li D, Qiu H, Cao X (2022) Evaluation of long−term carbon sequestration of biochar in soil with biogeochemical field model. Sci Total Environ 822:153576 35104525 10.1016/j.scitotenv.2022.153576

[CR68] Zhang A, Cui L, Pan G, Li L, Hussain Q, Zhang X, Zheng J, Crowley D (2010) Effect of biochar amendment on yield and methane and nitrous oxide emissions from a rice paddy from Tai Lake plain, China. Agr Ecosyst Environ 139:469–475

[CR900] Zimmerman AR, Gao B, Ahn MY (2011) Positive and negative carbon mineralization priming effects among a variety of biochar-amended soils. Soil Biol Biochem 43(6):1169–1179

[CR69] Zub HW, Brancourt-Hulmel M (2010) Agronomic and physiological performances of different species of Miscanthus, a major energy crop. A review. Agron Sustain Dev 30:201–214

