# Supplementary material for: Wetter farming: raising water table and biochar for reduced GHG emissions while maintaining crop productivity in agricultural peatlands
Source: Biochar. 2025 Sep 15;7(1):110. doi: 10.1007/s42773-025-00487-7 (PMC12433923; doi:10.1007/s42773-025-00487-7)
Supplement: Supplementary file 1 — Supplementary material 1. [file 42773_2025_487_MOESM1_ESM.docx]

**Wetter farming: Raising water table and biochar for reduced GHG emissions while maintaining crop productivity in agricultural peatlands**

Peduruhewa H. Jeewani^a*^, Emmanuella Oghenefejiro Agbomedarho^a^, David R. Chadwick^a^ Chris D. Evans^b^, Davey L. Jones^a^

**(a)**

**(b)**


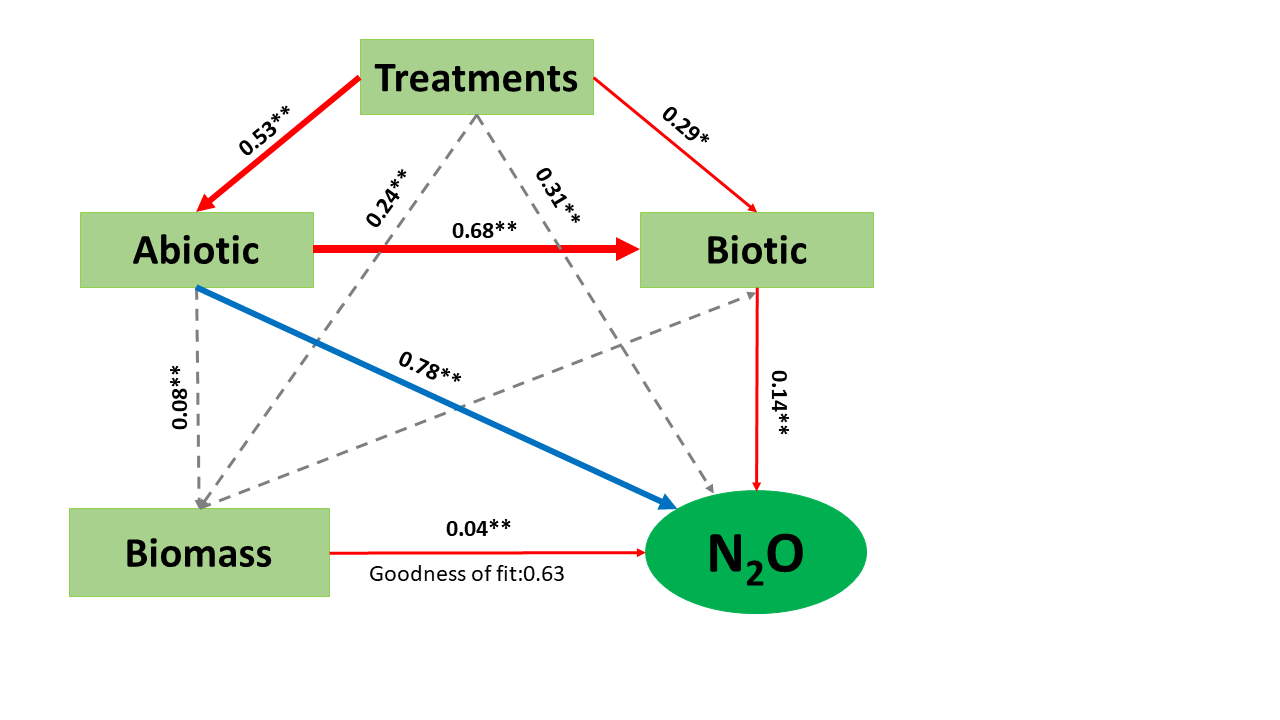

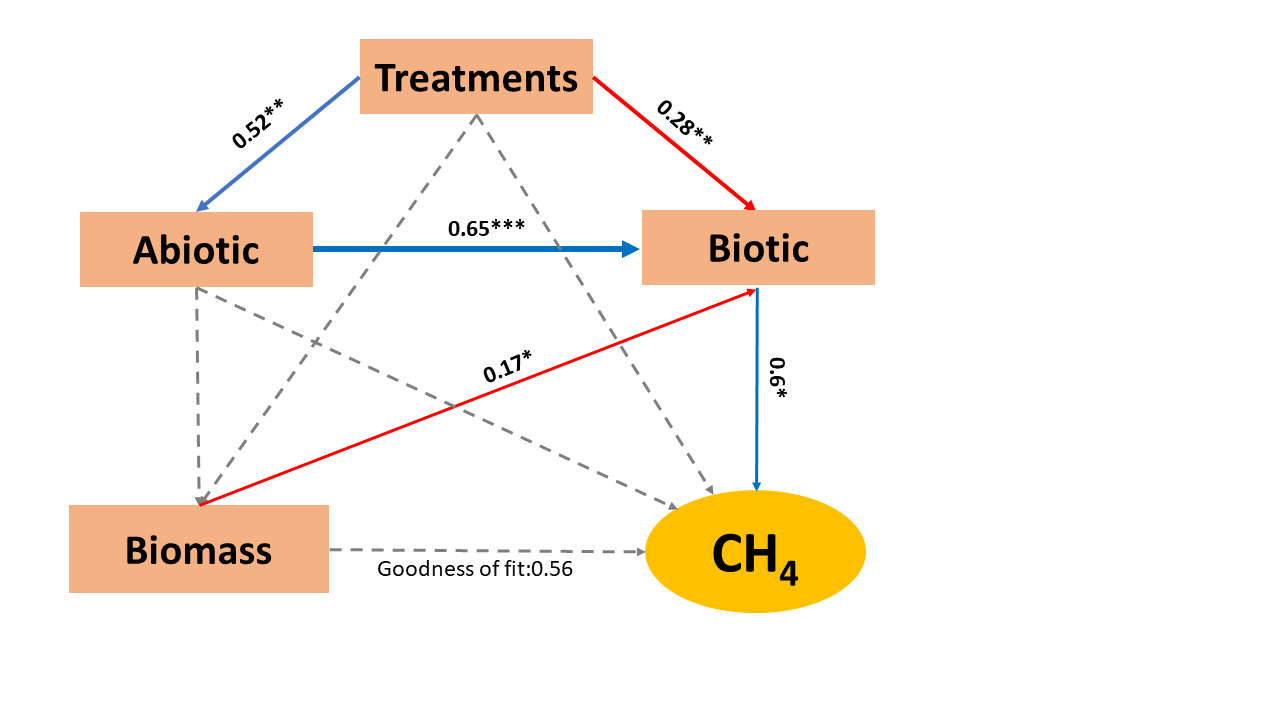

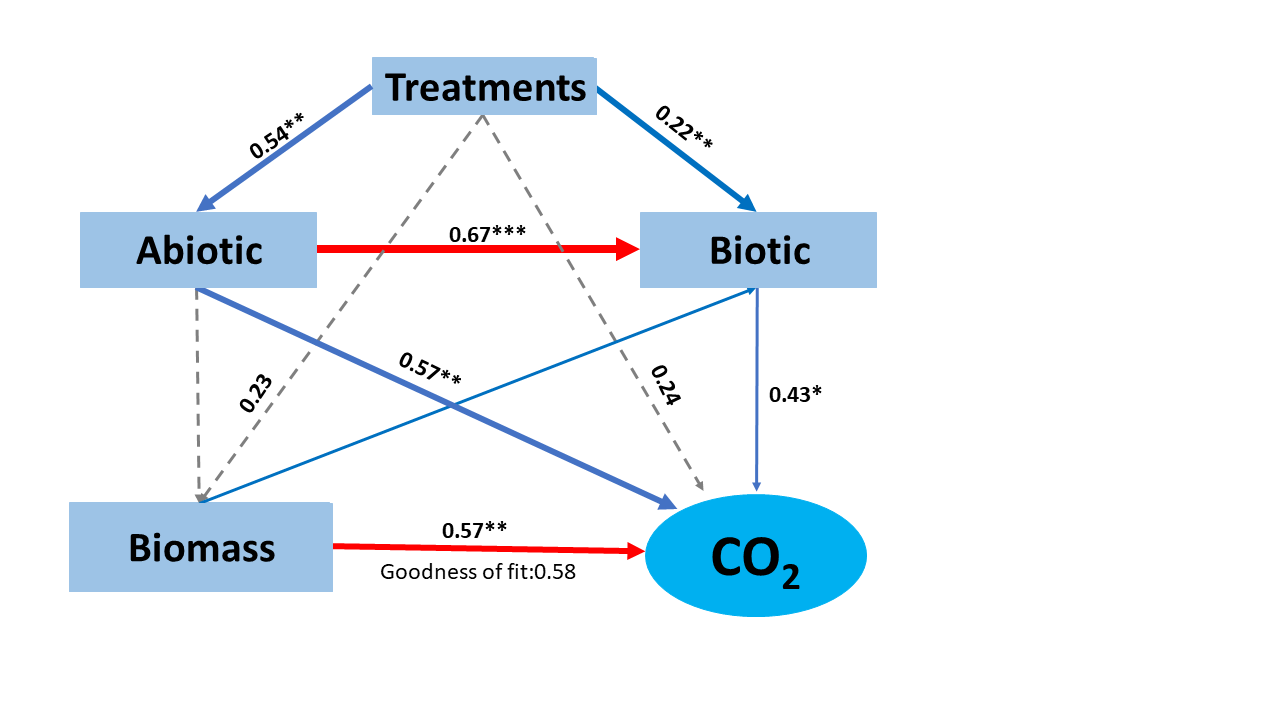


**(c)**


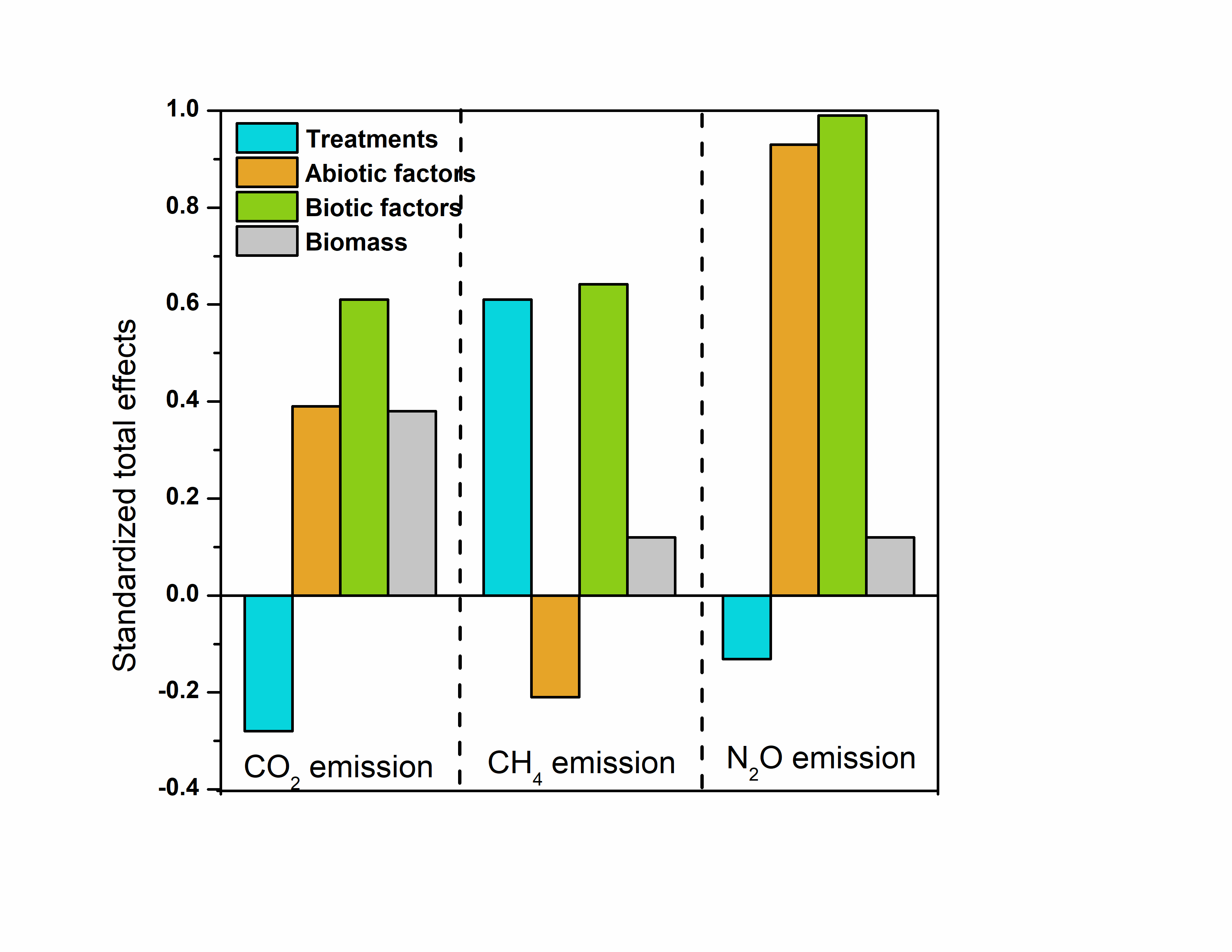


**(d)**

**Fig. S1** Partial least squares path modelling (PLS-PM) analysis of the relationships between treatments (biochar and water table levels -10cm and -15 cm), abiotic (pH, EC and temperature), biotic (fungal and bacterial Shannon index) and biomass (root and shoot biomass) on CO_2_ (a), CH_4_ (b), N_2_O (c), and Standardized total effects of treatments, abiotic and biotic properties on GHG (CO_2_, N_2_O and CH_4_) emission (d). 1000 bootstraps were conducted to estimate the path coefficients. Positive and negative effects are represented by blue and red arrows, respectively. Path coefficients that were not significantly different from zero are shown as grey dashed lines; *p < 0.05, **p < 0.01, and ***p < 0.001. Percentages above the boxes represent the explanatory power of the variables. The goodness-of-fit was used to assess the model.











**Fig. S2.** Effect of biochar amendments and water table management (HW -10 cm, and LW -15 cm from the soil surface) on the temporal variation of EC (a), pH (b) and temperature (c) in soil solution of an agricultural peat soil under lettuce production. The biochar amendments included *Miscanthus* biochar. Values represent means ± standard errors (*n* = 4). LW indicates water table level at -15 cm depth; HW indicates water table level at -15 cm depth.


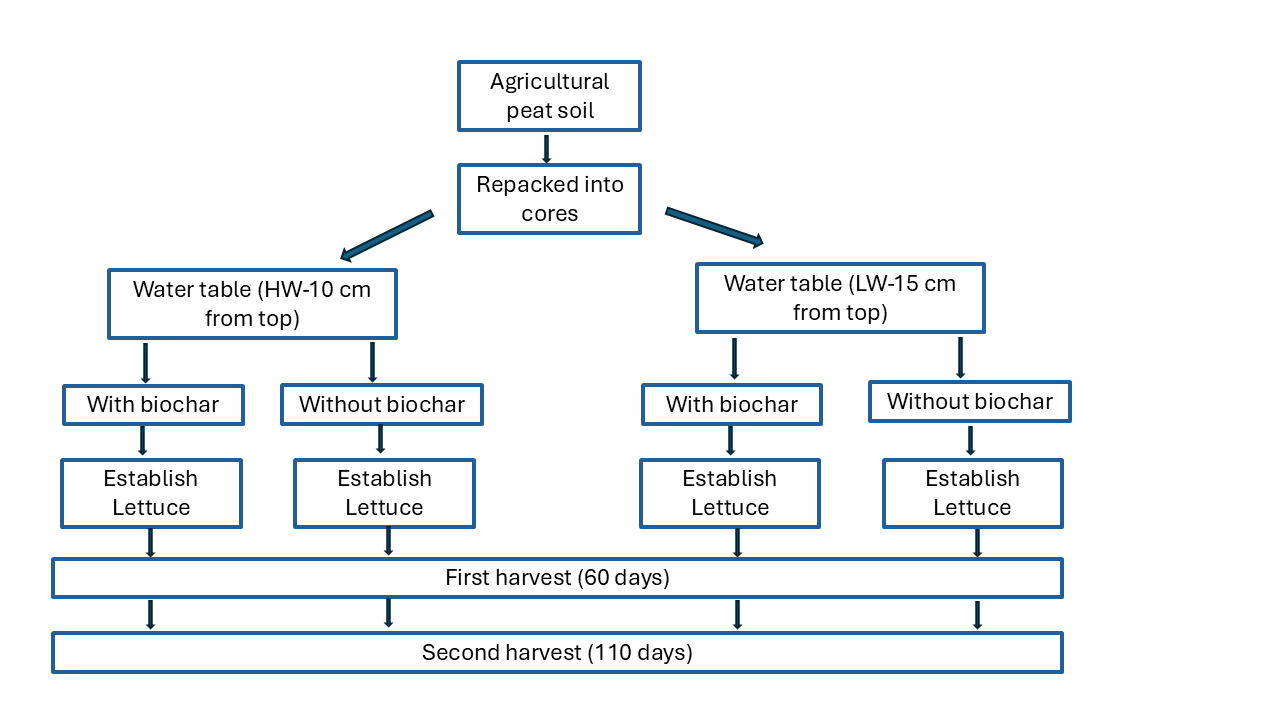


**Fig. S3.** Schematic diagram of experimental set up.

**Table S1:** Cumulative CO_2_ emissions per treatment during the whole experimental period.

| Sampling days | Biocha+HW  (g CO_2_ m^-2^) | Biochar+LW  (g CO_2_ m^-2^) | | | Control+HW  (g CO_2_ m^-2^) | | | Control+LW  (g CO_2_ m^-2^) | |
| --- | --- | --- | --- | --- | --- | --- | --- | --- | --- |
| 3 | 1.86±0.21 | | | 2.33 ± 0.74 | | | 1.19 ± 1.06 | | 2.63 ± 0.86 |
| 6 | 16.84 ± 1.56 | | | 20.85 ± 4.76 | | | 17.83 ± 5.61 | | 20.88 ± 6.53 |
| 8 | 23.38 ± 2.37 | | | 28.71 ± 6.25 | | | 26.68 ± 6.3 | | 29.43 ± 8.8 |
| 10 | 29.05 ± 3.19 | | | 35.32 ± 7.64 | | | 33.86 ± 7.03 | | 37.74 ± 11.21 |
| 13 | 39.27 ± 4.4 | | | 46.12 ± 10.04 | | | 46.2 ± 8.46 | | 52.8 ± 15.88 |
| 15 | 52.59 ± 5.93 | | | 60.75 ± 12.48 | | | 62.2 ± 10.15 | | 73.73 ± 21.85 |
| 17 | 63.06 ± 6.99 | | | 70.82 ± 13.07 | | | 75.24 ± 11.61 | | 89.44 ± 25.89 |
| 20 | 78.59 ± 8.55 | | | 87.08 ± 13 | | | 95.64 ± 14.25 | | 110.95± 30.43 |
| 23 | 94.26 ± 10.68 | | | 110.65 ± 13.05 | | | 120.77 ± 18.11 | | 138.32± 38.44 |
| 27 | 121.27 ± 17.45 | | | 150.55 ± 13.17 | | | 164.8 ± 26.47 | | 183.82 ± 53.78 |
| 30 | 156.67 ± 24.8 | | | 197.12 ± 11.47 | | | 212.24 ± 34.2 | | 231.13 ± 67.08 |
| 34 | 225.15 ± 34.08 | | | 280.13 ± 8.6 | | | 291.01 ± 45.65 | | 311.42 ± 82.7 |
| 37 | 298.43 ± 44.94 | | | 366.29 ± 13.93 | | | 381.77 ± 58.62 | | 411.65 ± 94.65 |
| 42 | 408.74 ± 60.66 | | | 490.93 ± 35.13 | | | 525.98 ± 71.38 | | 605.26± 112.9 |
| 45 | 479.02 ± 69.2 | | | 572.73 ± 39.22 | | | 616.04 ± 76.34 | | 724.71± 127.11 |
| 51 | 588.84 ± 81.42 | | | 706.11 ± 35.3 | | | 758.24 ± 86.12 | | 889.51 ± 148.21 |
| 57 | 727.21 ± 91.89 | | | 861.67 ± 40.48 | | | 931.28 ± 92.87 | | 1025.91 ± 143.39 |
| 60 | 781.59 ± 98.71 | | | 911.24 ± 44.38 | | | 1003.44 ± 95.82 | | 1088.46 ± 135.32 |
| 65 | 835.57 ± 106.96 | | | 952.31 ± 45.48 | | | 1079.57 ± 101.2 | | 1179.63 ± 136.34 |
| 75 | 904.61 ± 107.38 | | 1028.95 ± 47.84 | | | 1181.54 ± 104.13 | | | 1263.45 ± 142.47 |
| 85 | 927.43 ± 108.74 | | 1068.35 ± 49.51 | | | 1231.54 ± 101.23 | | | 1447.28 ± 143.41 |
| 95 | 1001.22 ± 114.55 | | 1105.84 ± 50.69 | | | 1243.32 ± 108.7 | | | 1547.06 ± 145.64 |
| 110 | 1031.22 ± 124 | 1205.84 ± 51 | | | 1343.32 ± 98.7 | | | 1647.06 ± 148.64 | |
